# Storm surge hydrographs from historical observations of sea level along the Dutch North Sea coast

**DOI:** 10.1007/s11069-025-07351-8

**Published:** 2025-05-29

**Authors:** Mia Pupić Vurilj, José A. Á. Antolínez, Sanne Muis, Oswaldo Morales Napoles

**Affiliations:** 1 Department of Hydraulic Engineering, Delft University of Technology, Delft, Netherlands; 2 Institute for Environmental Studies, Vrije Universiteit Amsterdam, Amsterdam, Netherlands; 3 Hydrodynamics and Forecasting Department, Deltares, Delft, Netherlands

**Keywords:** Extreme sea levels, Storm surge, Event type, Tide gauge records, Consecutive storms, Clustering

## Abstract

Due to changing climates and rising sea levels, low-lying coastal regions, such as the Netherlands, face increased risks of flooding driven by extreme sea levels. Thus, understanding extreme sea level events and their underlying dynamics is crucial for effective coastal management. This study developed and applied a novel classification framework to investigate historical storm surge events along the Dutch coast and improve the understanding of regional storm surge dynamics. Using 16 sea level records, storm surges were identified with the Peak Over Threshold (POT) method, using the 70th (POT70) and 99th (POT99) percentiles as thresholds. POT70 captured a more comprehensive storm surge activity, including multiple peaks and successive surges that are critical for coastal management. In contrast, POT99 captured surge peaks but missed significant pre- and post-storm surge activities. The POT70-derived surges were classified into 56 event types using clustering methods based on surge values across the whole event time series, and event duration. Event types were then characterised by temporal patterns, peak magnitude, duration, probability of occurrence, yearly frequency, and cumulative surge intensity. Key findings revealed frequent two-peak storm surges and significant variations in storm surge intensity along the coast, with stronger events occurring in northern regions. The results highlight the complexity of storm surge patterns, indicating that while simplified hydrograph models are useful, they may not always capture the full range of surge pattern variations. This novel classification framework offers a more detailed approach to evaluating surge patterns and can be applied to other coastal regions as well.

**Supplementary Information:**

The online version contains supplementary material available at 10.1007/s11069-025-07351-8.

## Introduction

Due to changing climates and rising regional sea levels, what were once considered rare extreme sea level events, occurring once per century, are expected to become annual occurrences at over half of all tide gauge locations by the end of the twenty-first century (IPCC 2021). This poses an increasing risk of coastal flooding for low-lying coastal and island regions, including those with established protections like the Netherlands, the United States, or Indonesia (Kron 2013). Therefore, traditional engineering design, which relies on statistical extrapolation of present natural variability, must evolve in response to these uncertainties. Future solutions should combine probabilistic methods, machine learning, and numerical modelling to better understand and predict extreme sea levels. Historic events, such as the North Sea Flood of 1953, should serve as stark reminders of the vulnerabilities these areas might face (e.g., Baxter 2005; Gerritsen 2005).

Extreme sea levels are composed of mean sea level, astronomical tide, storm surge, and wind waves (Muis et al. 2023) and are forced by low-pressure systems, strong winds, and local bathymetry (Arns et al. 2020; Merrifield et al. 2013). In estuarine areas, river runoff can also become a significant component of extreme sea levels (Lee et al. 2019; Kew et al. 2013). Therefore, along with sea level rise, local extreme sea levels are also influenced by regional dynamics and climate variability (Batstone et al. 2013; Rueda et al. 2017; Rueda et al. 2018). In this context, "regional" refers to broad geographic areas spanning multiple coastal zones, while "local" refers to site-specific variations at a smaller scale.

Most extreme sea level studies for the North Sea have primarily focused on peak magnitudes of individual events (e.g. de Valk and van den Brink 2023; Ganske et al. 2018; Dangendorf et al. 2016). However, there is a growing recognition of the importance of examining their temporal characteristics, such as the duration, clustering of storms, and the broader context of storm patterns (Haigh et al. 2016; Stephens et al. 2020; Anderson et al. 2019; Jenkins et al. 2023). For example, studies have shown that extra-tropical cyclones tend to cluster along the flanks and exit region of the North Atlantic storm track, especially during periods of increased cyclonic weather regimes (e.g. during positive North Atlantic Oscillation) (Hauser et al. 2023; Dacre and Pinto 2020). Successive storms can increase a region’s vulnerability, as disaster reduction resources are depleted in the face of repeated impacts (van den Brink et al. 2017). A recent study (Bakker et al. 2025) on the Maeslant Storm Surge Barrier in Rotterdam emphasised the importance of considering multiple storm surge peaks in flood risk management. The study's results suggested that compound events involving moderate extremes could have a major impact on flood risk. However, the analysis was limited to double-peak storms, excluding more complex scenarios. Additionally, previous research has shown that extended periods of elevated sea levels and persistent exposure to incoming waves can affect coastal environments and their resilience, resulting in greater erosion of beach-dune systems or changes in coastal habitat dynamics (Van Dijk 1994; van de Pol et al. 2010; Dodet et al. 2018; Dissanayake et al. 2015; Nieuwhuis et al. 2023).

A common approach in modelling and decision-making is representing extreme sea level events with a simplified triangular hydrograph of the associated storm surge. This method focuses on parametrising statistically extreme surge values and their above-threshold durations. However, focusing only on statistically high values may oversimplify the complexities of storm surge events and overlook crucial details such as the duration of elevated sea levels, multi-peak surge patterns, and post-storm surge conditions (Dullaart et al. 2022; Chbab 2015; Anderson et al. 2019; Serafin and Ruggeiro 2014; Martzikos et al. 2023; Duo et al. 2020; Vousdoukas et al. 2016). Based on modelled data, Geerse et al. (2019) showed that irregular storm surge hydrographs provide a more accurate representation of surge evolution along the Dutch coastline. However, they did not consider multiple-peak events, and such details are essential for fully understanding the impact of an event.

Therefore, there is a need for deeper physical insights into storm surge behaviour, particularly given the impacts of climate change. Recent advances in data analysis techniques, such as clustering methods, provide powerful tools to uncover patterns in storm surge events and their recurrence (Camus et al. 2024; Rueda et al. 2017; Enriquez et al. 2020; Li et al. 2023). This can improve our understanding of extreme event characteristics, refine modelling approaches, and support better coastal management decisions, including tasks like maintaining storm surge barriers or planning beach nourishment (van den Brink et al. 2017).

To address this need, this study developed and applied a novel classification framework to analyse historical storm surge events accounting for broader hydrograph behaviour. Sixteen sea level records were used to investigate various characteristics of historical non-tidal residual (NTR), or storm surge, events along the Dutch coast. These events were clustered into distinct types and characterised by temporal patterns, peak magnitudes, duration distributions, probabilities of occurrence, yearly frequencies, and cumulative surge intensities. The study begins with a short description of the study area, followed by a detailed outline of the materials and methods used to define and classify storm surge events. The most common types of storm surge events across the coast are identified, and some historical events are highlighted. Next, the results are presented, offering a characterization of these event types. The discussion section provides an interpretation of the findings, exploring their implications, relevance, and limitations. Finally, the key findings are summarized, and some recommendations for future steps are noted.

### Study area

The Dutch coastline, located along the southeastern edge of the North Sea, is a low-lying region that is highly vulnerable to extreme sea level events. About 26% of the country lies below mean sea level, and about 60% is vulnerable to floods. The coast consists of sandy beaches, dunes, tidal flats, estuaries, and barrier islands, all of which play a key role in natural flood defence. However, these features are increasingly threatened by sea level rise, extra-tropical cyclones, and ongoing land subsidence (Mulder et al. 2011).

The bathymetry of the North Sea, with its broad and shallow continental shelf, plays a crucial role in influencing tidal patterns, surge propagation, and wave behaviour along the Dutch coast (Otto et al. 1990). The astronomical tide system follows a semi-diurnal cycle, but tidal ranges vary significantly. The southern region of the Netherlands around Zeeland experiences the largest tidal ranges, with values ranging from 1.9 to 4.7 m at Westkapelle in 2024. From there it gradually decreases, reaching between 0.7 and 2.1 m at Den Helder. To the east of Den Helder, the tidal range increases again, with values of 1.1 to 3.0 m at Nes in 2024 (Rijkswaterstaat Getij 2024). This variation is driven by regional topography, geometry, and the location of the amphidromic points in the North Sea. The main tidal constituents are the M2 (principal lunar semidiurnal) and S2 (principal solar semidiurnal).

Atmospheric conditions over the Netherlands are heavily influenced by its temperate maritime climate, with weather patterns shaped by prevailing westerly winds and frequent Atlantic low-pressure systems. These systems often bring strong winds and heavy rainfall, particularly during autumn and winter. Along the coast, north and north-westerly low-pressure systems generate the most severe storm surges due to the long wind fetch (van den Hurk et al. 2015). Moreover, these patterns strengthen during a positive phase of the North Atlantic Oscillation (NAO) and the North Sea experiences stronger and more frequent cyclones (Hurrell 1995; Hurrell et al. 2003; Dangendorf et al. 2014). 

When storm surges coincide with high tides, they pose a significant threat to the Dutch coast. The southwestern delta region, where the Rhine, Meuse, and Scheldt rivers converge with the sea, is particularly vulnerable to flooding due to the risk of compound events with high river flows and storm surges (Zijl et al. 2013; van den Hurk et al. 2015, Kew et al. 2013). During such events, it may be necessary to open the coastal flood defences to allow river discharge to flow into the sea. Further north, the Wadden Sea, with its tidal flats and barrier islands, also faces considerable risk from storm surges. These areas rely heavily on natural features like dunes and sandbanks for protection (Brand et al. 2022).

To mitigate these risks, the Netherlands has implemented extensive infrastructure, such as the Delta Works system of storm surge barriers, dikes, and sluices (Delta Act 1958; Flood Defense Act 1996; Water Act 2009). However, climate change challenges these measures. While engineers can adapt to gradual sea level rise by heightening the coastal defences, the added impacts of compound events, increased winter precipitation (van der Wiel 2024) and salinisation (Deolu-Ajayi et al. 2024) require ongoing adaptation.

## Materials and methods

**Fig. 1:**
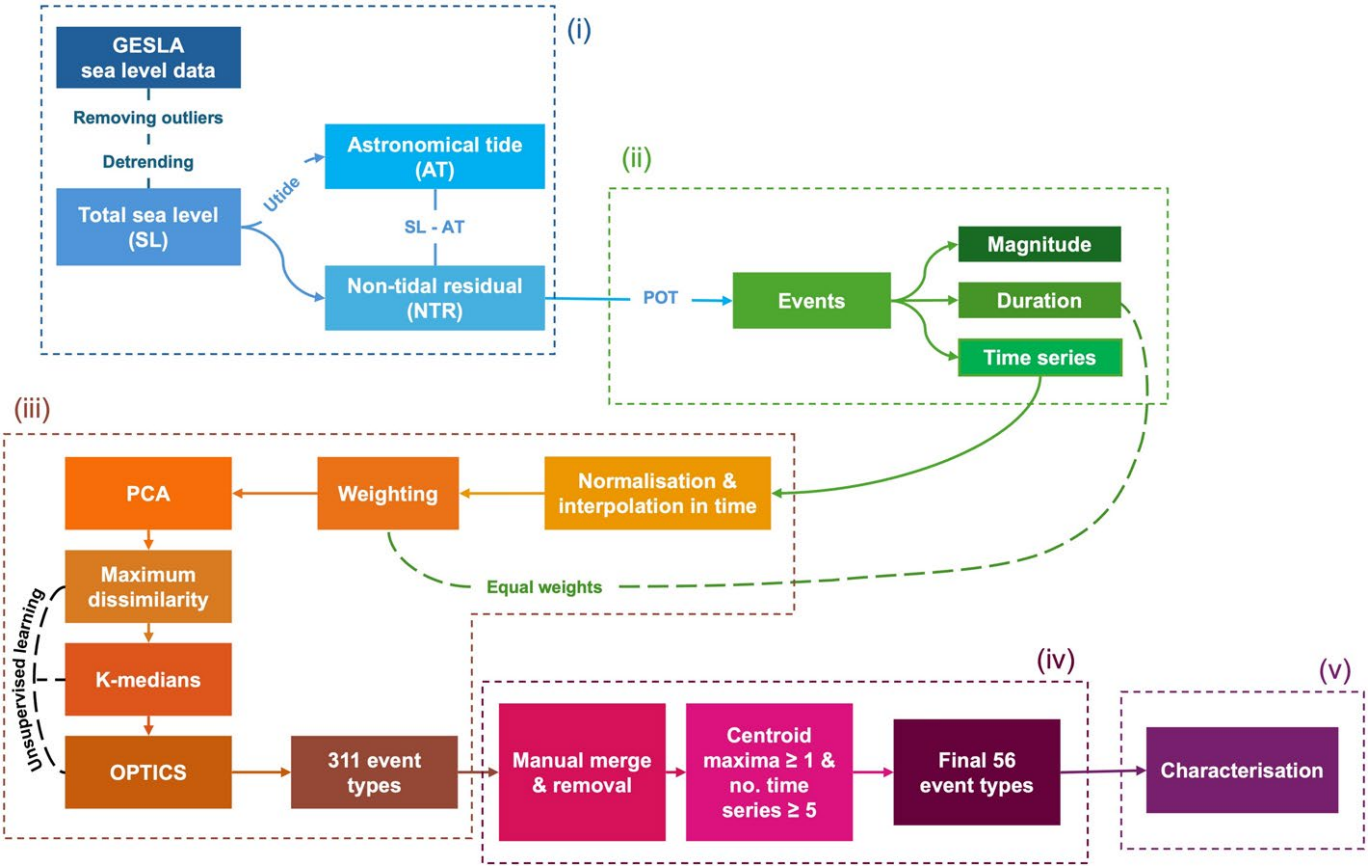
Flowchart illustrating the methodology of the study, detailing the five key stages: **i** preprocessing of observed sea level data, **ii** extraction of storm surge events, **iii** preparation and clustering of time series, **iv** refinement of event types, and **v** characterisation of event types

This study’s methodology can be divided into 5 parts, as schematised in Fig. 1.

### Preprocessing of observed sea level data

**Fig. 2:**
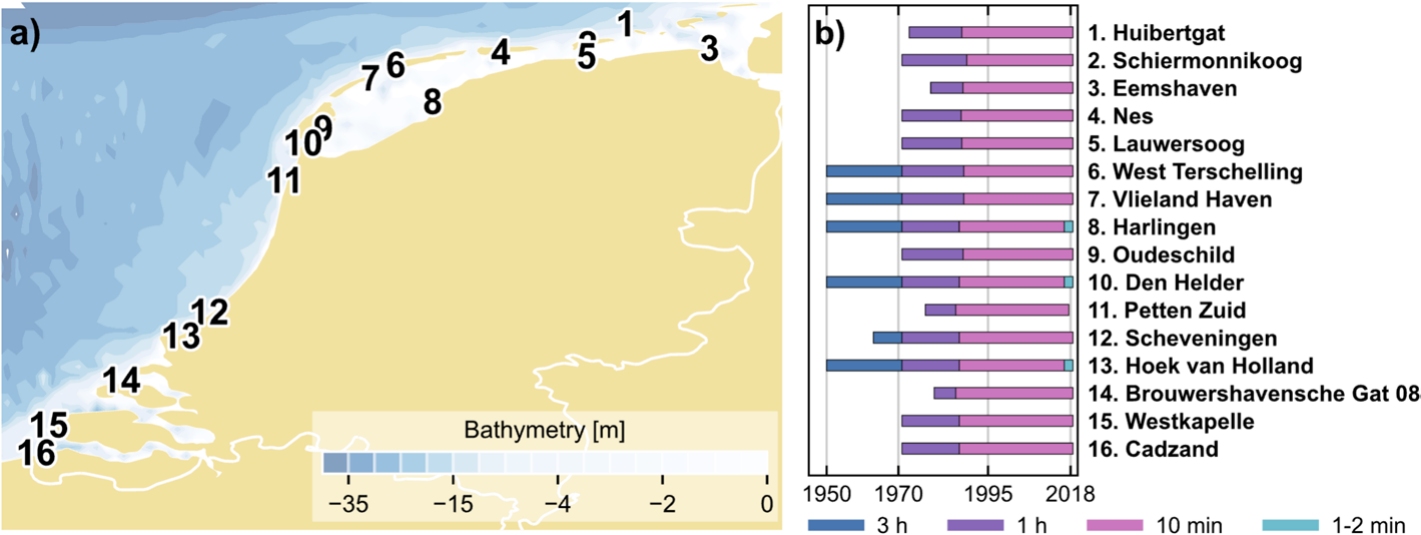
**a** Map and bathymetry (GEBCO Compilation Group 2024) of the Dutch coast, showing the location of the sixteen GESLA-3 stations used in this study and **b** the duration of the sea level records with frequency periods

Observed sea level data were used to gain a better understanding of observed storm surge dynamics and validate model estimates of storm surge reanalysis and forecast. This is essential for climate change assessments where reanalysis data serves as a benchmark for climate models. The data were collected at tidal gauges owned by Rijkswaterstaat (Rijkswaterstaat Waterinfo 2024), provided within the GESLA-3 (Global Extreme Sea Level Analysis) dataset (Haigh et al. 2023). Sixteen stations along the Dutch coast were selected, with observational periods ranging from 38 to 68 years and observation frequencies varying over time (Fig. 2).

The observed total sea level was searched for outliers, linearly detrended to remove long-term trends caused by sea level rise, and then split into two components: (i) the tidal component, and (ii) the non-tidal residual (NTR). The former was derived using harmonic tidal analysis (UTide python package by Wesley Bowman 2014; configuration is detailed in the Supplementary Information (SI)**)** and the latter was calculated by removing the predicted tidal signal from the observed total sea level. The NTR thus includes water level variations caused by meteorological factors, potentially some wave effects, river runoff, and the non-linear interactions between them. Since the tidal phase can be affected by depth changes due to the surge, and vice versa, the NTR also includes the non-linear effect of tide-surge interaction. The data remained in its original frequency, and any gaps were not filled.

### Extraction of storm surge events

Events were identified using the peak over threshold (POT) method on NTR, with a 70th percentile threshold per station (referred to as POT70). First, all values exceeding the threshold were identified. If two exceedances occurred within three days, they were merged into a single event with only the higher peak value retained for further comparison. This process was repeated iteratively ensuring a 3-day independence gap between events. From here on, these events are referred to as *storm surges*, and the time elapsed between the first and last threshold-exceeding moment is referred to as *event duration*. The relatively low threshold (POT70) was used to extend the duration of statistically extreme events, capturing the full storm surge development rather than focusing solely on peak sea level magnitudes. Once identified, the events were filtered by storm surge height, enabling a focus on more extreme cases while preserving hydrograph details. However, using the initial low threshold allows future studies to explore moderate events, which could still be relevant for beach processes and coastal maintenance. This approach resulted in a total of 45,843 events across stations.

Similarly, statistically extreme events were extracted as well, using a 99th percentile threshold per station (referred to as POT99). These events were compared with those identified using POT70 to evaluate differences and validate the results.

### Preparation and clustering of time series

Commonly, storm surge events are parametrised before clustering (Camus et al. 2021, 2024; Rueda et al. 2017); however, in this method, storm surge values across the whole time series were clustered. The POT70 storm surges were used, and a multi-step process was undertaken to prepare and analyse the data through unsupervised clustering of the time series:

I. *Normalisation*: Normalising storm surge time series in time to ensure comparability across different events. The index of each time series was normalised to range between 0 and 1, based on the total duration of the series in hours.
II. *Resampling the time axis*: Resampling the time series to achieve uniform time intervals with the event featuring the largest time step and duration combination used as the reference. Linear interpolation was applied to align all other events with this uniform grid. The reference event for this interpolation occurred from 8 Jan 16:40 to 24 Jan 07:40, 1956 at West Terschelling, lasting 375 h with a time step of 3 h. As a result, all time series were interpolated to a uniform set of 1,125 regular time steps.
III. *Feature augmentation*: Adding the duration of each event as an additional feature to the clustering process. Now we have the following features for clustering: the time series (1,125 surge values) and the event duration (in hours).
IV. *Standardisation*: Standardising both time series and duration features to ensure they are on the same scale. Each resulting feature has a mean of 0 and a standard deviation of 1.
V. *Variance weighting*: Weighting the time series and duration features so that each account for 50% of the total variance in principal component analysis (PCA).
VI. *Dimensionality reduction*: Applying PCA to the weighted features to reduce data dimensionality, retaining 99% of the total variance. This reduced the number of features from 1,126 to 62.
VII. *Maximum dissimilarity sampling*: Using a maximum dissimilarity algorithm (MDA) (Kohonen 1982) to select a subset of 10% of the total data (4,584 events) to ensure a representative sample. This initial subsetting is required to avoid known issues with sample density in clustering techniques, which guarantees that less frequent events are well represented in the clustering process (Camus et al. 2011; Athanasiou et al. 2021; Scott et al. 2020).
VIII. *Initial clustering*: Clustering of the MDA subset into 400 clusters using the K-medians algorithm (Wittek 2014), applying K-means + + as the initialization step.
IX. *Centroids clustering*: Further clustering of the 400 centroids using the “Ordering Points To Identify the Clustering Structure” (OPTICS) (Ankerst et al. 1999) algorithm to merge some clusters, resulting in 311 distinct clusters, i.e. event types.

It is important to note that the clustering method does not distinguish between the 16 stations. As a result, an event driven by the same forcing may be classified under different event types for different stations. This occurs because environmental conditions vary across stations, potentially causing the same forcing to generate distinct surge patterns at each site.

A more detailed description of the methods can be found in SI.

### Refinement of event types

Based on the self-organising maps (SOM) topology (Kohonen 1982; Camus et al. 2011), a visual inspection of the resulting 311 event types was conducted. The clusters were sorted by centroid (median of all time series in a cluster) similarity, and events exhibiting closely matching surge patterns (very similar amplitude and timing) were merged. On the other hand, events characterised by patterns that did not resemble surge dynamics, such as a low constant surge due to the low 70th percentile threshold, were excluded. The remaining event types were then filtered based on the peak magnitude of their cluster centroids, retaining only those with a peak magnitude exceeding 1 m, corresponding to around the 99th percentile of NTR values. Additionally, only event types with at least five associated time series were kept. A final visual examination of the clusters revealed no further need for merging. This process narrowed down the analysis to 972 time series belonging to 56 event types.

### Characterisation of event types

For each of the 56 event types, the probability of occurrence was computed as

1 $$\begin{array}{*{20}c} {P = \frac{n}{N},} \\ \end{array}$$

where $n$ is the number of time series within a cluster and $N$ = 972 is the total number of time series across all clusters.

The distributions of event durations and yearly frequencies were analysed as well. The yearly frequency of an event type was calculated by first identifying the date of the peak magnitude for each time series in that type. Peaks within a cluster that occurred within ± 2 days of each other were then grouped as a single event, accounting for the possibility that the event impacted different stations at different times. Each unique group of peaks was then counted as one event, regardless of how many stations recorded it. In this way, the distinct events that hit the coast were determined, avoiding multiple counts of the same event across different stations.

The percentage of each event type at each monitoring station was calculated to identify the most common types. For these prevalent event types, the probability of occurrence, yearly frequency, distribution of event durations, and the total time during which surge values exceeded the 99th percentile were further examined.

Moreover, the intensity of a storm surge was determined by integrating sea level values that exceeded the 99th percentile over the event's duration. The cumulative storm surge intensity ($I$) was thus calculated using the formula:

2 $$\begin{array}{*{20}c} {I = \mathop \smallint \limits_{{t_{i} }}^{{t_{f} }} NTR_{above} \left( t \right)dt,} \\ \end{array}$$

where $NTR_{above}$ represents the non-tidal residuals above the 99th percentile, $t_{i}$ denotes the initial time, and $t_{f}$ refers to the final time. The result is expressed in meter-hours (mh). To compute this, we used the composite trapezoidal rule.

Ten historical storms in the North Sea were selected for further analysis. We calculated the cumulative storm surge intensity for each event, ranked them, and chose the top five most intense events. The remaining five were selected using a 45 mh threshold (corresponding to the top fifty events), a list of major windstorms in the Netherlands from the Royal Netherlands Meteorological Institute (KNMI) (KNMI, 2024), and expert judgment. Therefore, these ten selected storms should not be interpreted as a comprehensive record of the highest total water level events. The selected historical storms are briefly described below, highlighting their geophysical characteristics and impacts, based on storm surge reports from the Rijkswaterstaat (Rijkswaterstaat 1948, 1998).

#### *Storm 1—1990 cyclones Vivian and Wiebke*

The most intense occurrence of this event in the dataset occurred from 23 Feb to 1 Mar 1990 at Harlingen. This event was caused by two consecutive low-pressure systems: Vivian and Wiebke. Vivian formed on February 25 and dissipated on February 28, coinciding with the formation of Wiebke. These two storms caused widespread damage and loss of life, particularly in Germany and Switzerland. The Oosterschelde storm surge barrier was closed four times during the event. According to KNMI, peak wind speeds were reached on 26 Feb, reaching 10 on the Beaufort scale, coming from the west-northwest (WNW).

#### *Storm 2—December 1954*

The most intense occurrence of this event in the dataset occurred from 17 to 26 Dec 1954 at Harlingen. This event was caused by two almost identical consecutive low-pressure systems, both tracking from Iceland to southern Sweden. At Harlingen, sea levels exceeded those recorded during the North Sea Flood of 1953. According to KNMI, peak wind speeds were reached on 21 Dec, reaching 10 on the Beaufort scale, coming from the northwest (NW).

#### Storm 3—January 1993

The most intense occurrence of this event in the dataset occurred from 21 to 28 Jan 1993 at Eemshaven. This event was caused by two active low-pressure systems that followed quickly. While the total water levels were not exceptional, the Oosterschelde storm surge barrier closed once, and the Hollandse IJssel barrier closed twice. Winds reached force 9–10 on the Beaufort scale from the WNW, though they were weaker in the southern coastal areas.

#### Storm 4—November 1977

The most intense occurrence of this event in the dataset occurred from 11 to 18 Nov 1977 at Nes. This event was caused by a combination of consecutive low-pressure systems: from Nov 11 to 13, two systems approached Norway and northern Denmark from the WNW, followed by two more from Nov 13 to 16, moving from Iceland across northern Denmark. According to KNMI, the peak wind speed was reached on 14 Nov, reaching 10 on the Beaufort scale. The winds ranged throughout the event from WSW to WNW.

#### Storm 5—November 1981

The most intense occurrence of this event in the dataset occurred from 18 to 27 Nov 1981 at Eemshaven. This event was caused by a low-pressure system that moved eastward on November 23, starting between Iceland and Scotland, and reaching the Norwegian west coast by the morning of November 24. During the 23rd, the wind was SW, which prevented the total sea levels from becoming extreme. From the 24th onward, the wind shifted to WNW. At sea, the wind reached force 11, but due to the initial wind direction, damage in the Netherlands remained limited.

#### Storm 6—2012 cyclones Ulli and Andrea

The most intense occurrence of this event in the dataset occurred from 1 to 8 Jan 2012 at Harlingen. This event was caused by two consecutive low-pressure systems, Ulli and Andrea, that moved across the northern North Sea from Scotland to Norway. Ulli formed on 31 Dec 2011, and dissipated on 7 Jan 2012, when it was absorbed by Andrea, which formed on 3 Jan and dissipated on 9 Jan. This caused a complex low-pressure system, leading to the closure of the storm barrier at Krimpen aan Den IJssel. Winds ranged from WSW to WNW and reached 8–9 on the Beaufort scale.

#### Storm 7—North Sea Flood of 1953

The most intense occurrence of this event in the dataset occurred from 25 Jan to 3 Feb 1953 at Hoek van Holland. This event was caused by a strong NW low-pressure system that formed south of Iceland and developed into a hurricane-force storm north of Scotland. According to KNMI, the peak wind speed was reached in the night of 31 Jan to 1 Feb, reaching 11 on the Beaufort scale. The winds ranged throughout the event from W to WNW and finally to NW. The event coincided with high tides causing extreme sea levels. The severe weather caused substantial damage, with dykes being breached and lives tragically lost. In response to this event, The Netherlands developed the Delta Works, an extensive system of dams and storm surge barriers.

#### Storm 8—2013 cyclone Xaver

The most intense occurrence of this event in the dataset occurred from 4 to 9 Dec 2013 at Eemshaven. This event was caused by a low-pressure system Xaver that formed south of Greenland on 4 Dec and rapidly deepened east across northern Scotland towards southern Scandinavia. According to KNMI, the peak wind speed was reached on 5 Dec, reaching 10 on the Beaufort scale, coming from the NW. The high astronomical tides led to record sea levels, with Vlissingen experiencing its highest sea levels since February 1, 1953, and Hoek van Holland seeing its highest levels since November 9, 2007. In response, the primary flood defences were closed at Den Oever, Harlingen, and Delfzijl, along with the Oosterschelde and Hollandse IJssel storm surge barriers.

#### Storm 9—Gale of January 1976

The most intense occurrence of this event in the dataset occurred from 27 Dec 1975 to 6 Jan 1976 at Harlingen. This event was caused by two low-pressure systems: one advancing from the Atlantic across northern Scotland toward southern Scandinavia, and another moving from southern Iceland, which was absorbed by the first one on 3 Jan. As a result, the eastward movement of the system was slowed down, causing the event to last longer. According to KNMI, the peak wind speed was reached on the night of 2 to 3 Jan, reaching 11 on the Beaufort scale, coming from the NW.

#### Storm 10—2007 cyclone Tilo

The most intense occurrence of this event in the dataset occurred from 1 to 12 Nov 2007 at Eemshaven. This event was caused by a NW low-pressure system Tilo. During the event, the closure level for the Maeslantkering was temporarily lowered, leading to its closure for the first time since its construction in 1998, along with the Hartelkering. The Oosterschelde storm surge barrier was closed as well. The resulting sea levels across the coastal area were high, with occurrences estimated to happen on average between 230 and 55 times per 1,000 years. Winds reached 8–9 on the Beaufort scale, coming from the NW.

### Other metrics

To assess the influence of the NAO on storm surge frequency, the correlation between the mean yearly NAO index and the annual count of storm surge events was analysed using Pearson’s *r*, Spearman’s *ρ*, and Kendall’s *τ*. The 972 storm surge events were aggregated per year across different clusters to determine the total yearly count.

The temporal relationship between storm surge peaks and the nearest high tide was also examined. First, the storm surge time series were processed using a 12-h moving average filter to reduce noise while preserving fluctuations. Peak detection was then performed on the smoothed signal using a prominence threshold of 0.02. Prominence measures how much a peak stands out relative to its surrounding valleys. Specifically, a peak’s prominence was determined by the vertical distance between a peak and the lowest contour level enclosing it without including a higher peak. Each peak was evaluated within a range of 48 h. Next, peaks were sorted by prominence and the highest ones were retained, enforcing a minimum separation of 12.5 h. Since smoothing may slightly shift peak locations, a final step refined the detection by searching for the true maxima in the unsmoothed time series within a ± 6-h window around each detected peak (example provided in Fig. SI4). This ensured that the selected peaks accurately correspond to actual high points in the data.

Finally, the phase difference between each extracted surge peak and the nearest high tide was calculated as:

3 $$\begin{array}{*{20}c} {\varphi = t_{peak surge} - t_{high tide} } \\ \end{array}$$

To visualise the result, the empirical Probability Density Function (PDF) and Kernel Density Estimation (KDE) were used. The KDE method provides a continuous probability distribution by smoothing discrete data points while avoiding assumptions about data distribution.

## Results

### Storm surge events

#### POT70

**Fig. 3:**
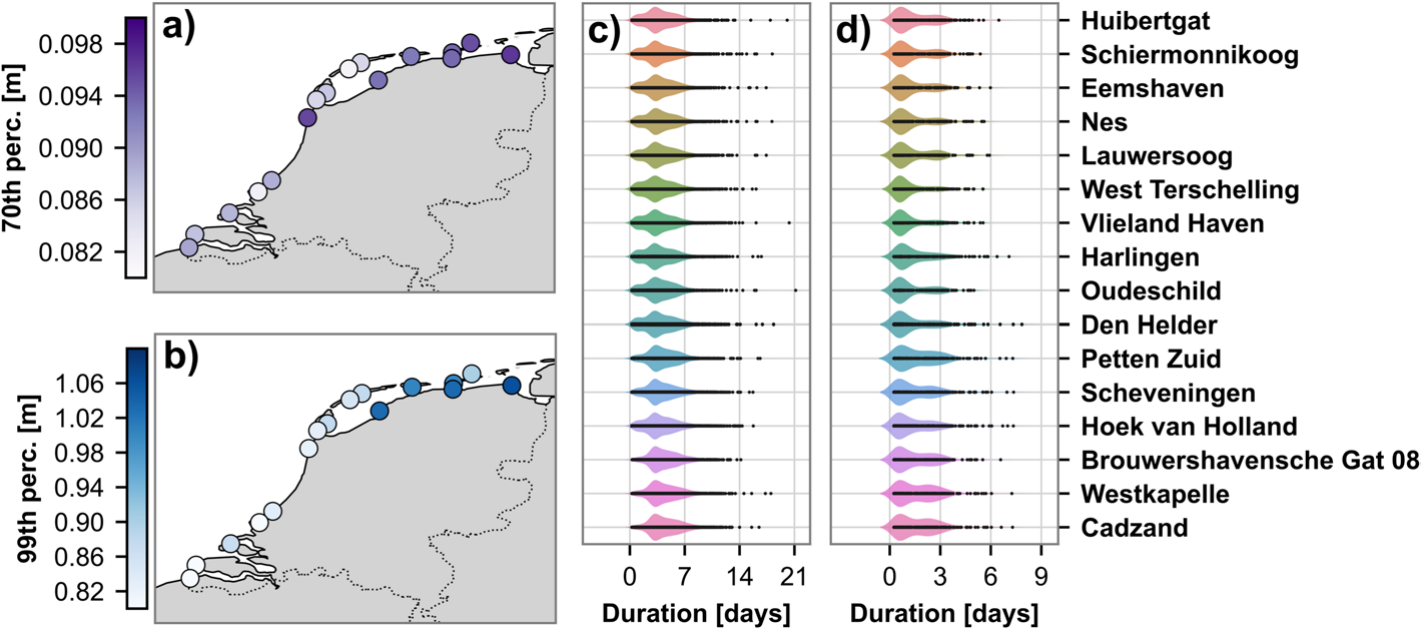
**a** The 70th percentile threshold per station, **b** the 99th percentile threshold per station, **c** the distribution of event duration per station for POT70, and **d** the distribution of event duration per station for POT99

The threshold for the POT70 of NTR was found to vary from 0.08 to 0.1 m along the coast (Fig. 3a). A total of 45,843 events were detected, with an average of 2,865 events per station. The distributions of the event’s peak magnitudes were similar across the coast (Fig. SI5a in SI), with slight deviations in the upper tails where the northern stations exhibited thicker tails. The distributions of event durations per station were broad, with some events exceeding 20 days (Fig. 3c). While the overall patterns were comparable, the stations in the south displayed a thinner lower tail. It is important to note that this duration was recorded for a low threshold and thus may also reflect storm arrival, dissipation, consecutive storms, and other more average conditions. Upon examining the time series, it was found that the POT70 threshold effectively detects multiple peaks and successive storm surge events.

#### POT99

The threshold for the POT99 of NTR was found to vary from 0.8 to 1.1 m along the coast (Fig. 3b) and a total of 4751 events were detected, with an average of 297 events per station. The distributions of events’ peak magnitudes varied across the coast (Fig. SI5b. in SI), with northern stations displaying broader distributions and peaks shifted toward higher values compared to southern stations. Harlingen stood out with a notably wider distribution than all other stations. The distributions of event durations per station exhibited a similar bimodal pattern (Fig. 3d). The first peak occurred around the 1-day mark, while the second peak varied between regions; the northern stations had a smaller second peak near 3 days, whereas stations south of Den Helder showed a more prominent second peak around the 2-day mark. The POT99-derived events focused on peak magnitudes and generally did not capture the storm surge development or successive events.

### Time series clustering

A total of 4584 time series were extracted by the MDA, ranging between stations as listed in Table 1 under the column ‘MDA subset’. The stations located on the eastern side of the Frisian Islands have the fewest selected events. The distributions of event durations and event’s peak magnitudes per each station showed a shift towards higher values, indicating that shorter and lower-peak-magnitude events were less frequently selected. Some spatial variations could be noticed as well, with northern stations having wider distributions, particularly noticeable for peak magnitudes (Fig. SI6 in SI).

**Table 1:** For each site, the table lists the two most common event types and the corresponding percentages. The "MDA subset" column indicates the number of time series recorded at each site after MDA, while the "Final number" column shows the total number of time series recorded at each station for the final 56 event types. Stations are arranged from north to south

Station	MDA subset	Final number	Prevalent clusters		Percentage	
			1st	2nd	1st	2nd
Huibertgat	149	38	1	3*, 7*	15.8	7.9
Schiermonnikoog	200	48	1	2, 3*, 4*, 5, 14, 15	16.7	6.3
Eemshaven	249	87	1	5	9.2	8.0
Nes	221	75	1	4*, 7*	10.7	6.7
Lauwersoog	359	88	1	14	14.8	6.8
West Terschelling	88	36	3*, 4*	1, 2, 5, 7*, 8, 14, 16	13.9	5.6
Vlieland Haven	62	22	2, 5	1, 7*	13.6	9.1
Harlingen	509	182	1	6*	11.5	6.6
Oudeschild	53	27	1, 4*, 5, 6*, 7*, 23	…	7.4	3.7
Den Helder	143	39	1	3*, 4*	15.4	7.7
Petten Zuid	465	53	4*	7*	11.3	9.4
Scheveningen	488	74	2, 8	1, 6*, 10, 11	8.1	6.8
Hoek van Holland	265	46	3*	4*, 6*	10.9	6.5
Brouwershavensche Gat 08	465	63	13	1, 6*, 9, 11, 19	9.5	7.9
Westkapelle	277	27	1	2, 3*, 6*	14.8	7.4
Cadzand	591	67	1	5, 8	13.4	9.0

*Two-peak event types

**Fig. 4:**
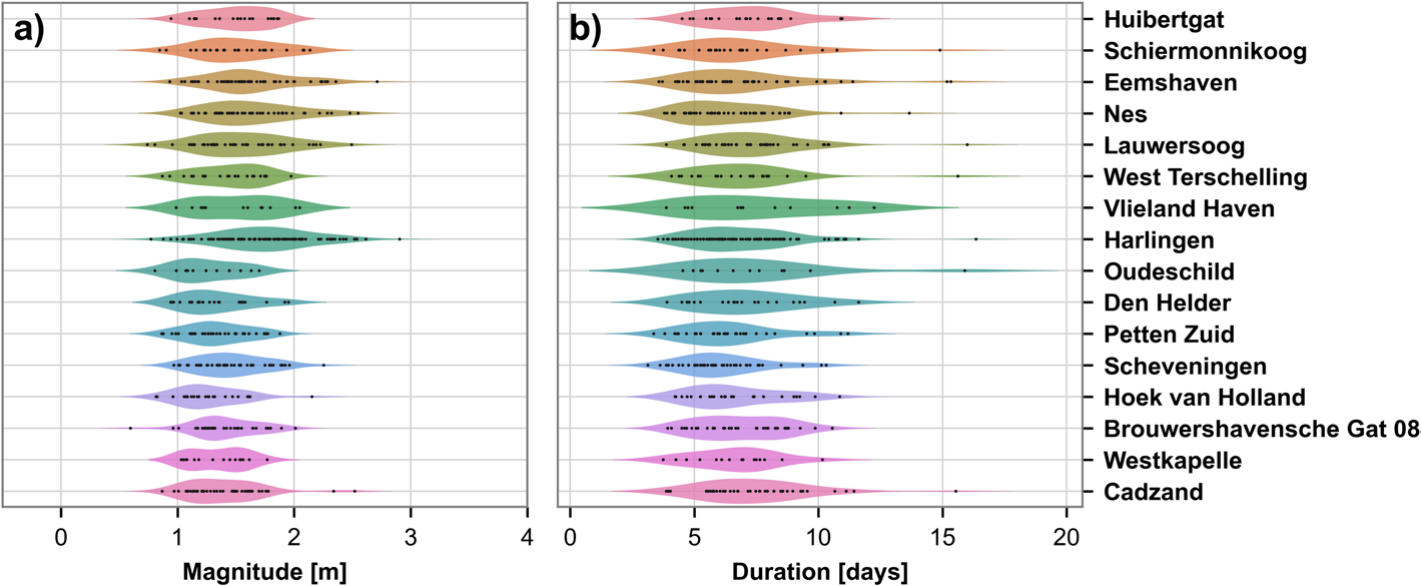
Distributions of **a** storm surge event’s peak magnitudes and **b** durations per station for the 972 time series classified into 56 event types

The clustering of the MDA time series subset identified a total of 311 distinct event types. However, the analysis was subsequently refined and filtered to focus on event types where the peak magnitude of the cluster centroid exceeded 1 m and had at least 5 related time series. This reduced the dataset to 972 time series across 56 event types for further detailed examination. The time series count per station is listed in Table 1, under the column ‘Final number’. The eastern-side Frisian Islands consistently had the fewest selected events, whereas Harlingen stood out with a significantly higher number of events compared to all other stations. The distributions of event durations and event’s peak magnitudes per station again showed a shift towards higher values and wider distributions in the northern regions (Fig. 4).

**Fig. 5:**
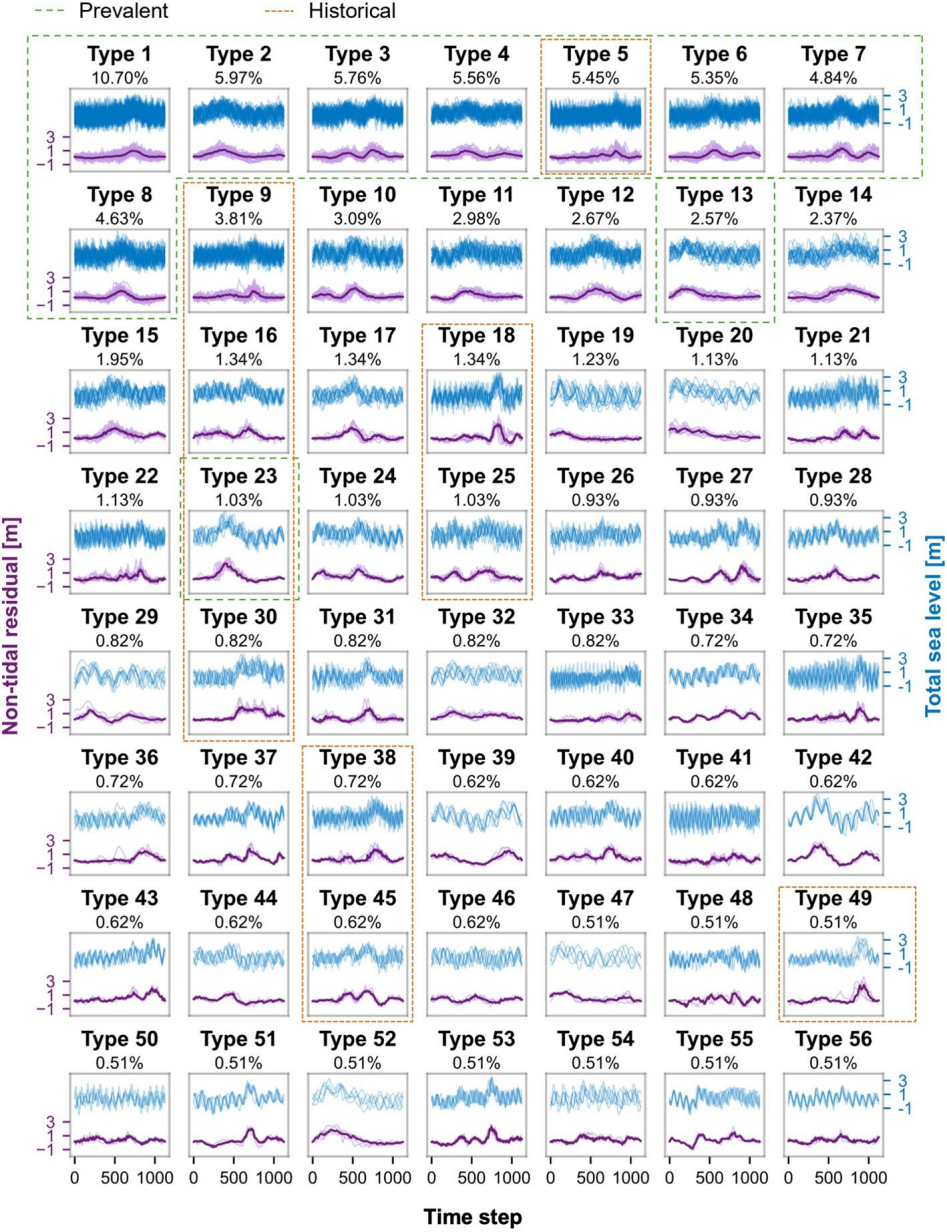
The 56 event types, ranked by the number of associated time series and probability of occurrence. For each type, all non-tidal residual time series are plotted at the bottom with the centroid highlighted in bold, while all total sea level time series are displayed at the top. The x-axis represents the interpolated time step, not a specific time unit. The most prevalent types are outlined with a dashed green square, and those containing the selected historical storms are outlined with a dotted orange square

Within the 56 selected event types (Fig. 5), a range of patterns was identified, including simple triangular shapes (e.g., Type 8), events with two or more peaks (e.g., Type 3, Type 27), as well as both short and abrupt types (e.g., Type 42) and longer types (e.g., Type 30). Notably, 56% of the centroids of these event types exhibited more than one distinct peak.

For each of these 56 event types, the probability of occurrence, yearly frequency, and event duration distributions were computed, with results presented in SI (Figs. SI7 and SI8). The duration distributions showed that normalised and interpolated time series with differences in duration of up to 186 h can exhibit similar shapes, as seen from Type 5. This occurs because, after interpolation, we directly compare the shapes of time series with different durations. Thus, even though the duration and time series shape were weighted equally, event durations within a cluster can vary. Despite this, the average duration width within a cluster is around 41 h.

### Characteristics of the most prevalent event types

Across the 16 sites considered, 10 specific event types emerged as the most prevalent (the top two most common types per site are listed in Table 1), with their rankings ordered by the number of associated time series (outlined with a dashed green square in Fig. 5).

Among the most prevalent event types identified, 40% exhibited centroids with two peaks (including Types 3, 4, 6, 7), observed at (from south to north) Hoek van Holland, Petten Zuid, Oudeschild, and West Terschelling. When examining the second most prevalent event types, it became clear that two-peak shapes were observed at nearly all stations, except for Cadzand, Lauwersoog, and Eemshaven. The exception could be due to the geographic and morphological characteristics of these stations, which are further discussed in the Discussion chapter.

**Fig. 6:**
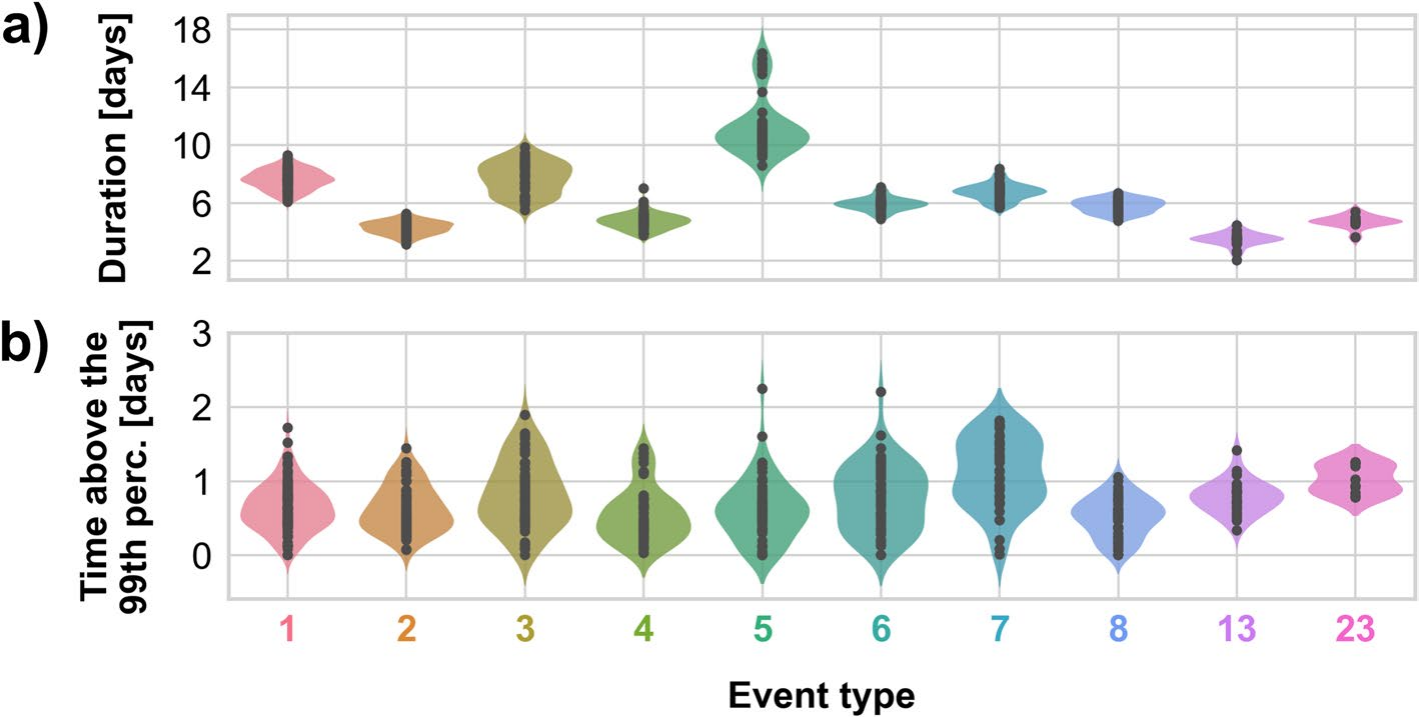
Distributions of the 10 most prevalent event types for **a** the total event durations, and **b** the total time during which the storm surge values exceed the 99th percentile

All four two-peak events exhibited durations within an acceptable range of about 75 h on average, as illustrated in panel a of Fig. 6. Interestingly, Type 5 had a bimodal distribution of duration, suggesting it could potentially be split into two distinct event types. However, because this event type started out slowly for all time series, likely due to the low 70th percentile threshold, and much of its duration was concentrated in this initial phase, it was kept merged. On the other hand, the distribution of total time intervals during which event values exceed the 99th percentile varied considerably (Fig. 6b). Events exceeding the 99th percentile for approximately 2 days displayed characteristics comparable to those with extreme sea level intervals extending only a few hours.

To understand the reason for the prevalence of events with two peaks, storm surge time series of Types 3, 4, 5, and 6 were examined in detail. In all cases, the second peak followed about two days after the first. Types 4, 6, and 7 had a higher first peak, with Type 7 showing the highest overall. These peaks typically occurred during low or rising tides. In contrast, Type 3 had a higher second peak, with the first occurring across various tidal conditions but still favouring rising tides. Overall, peak events across these types were most common during low or rising tides (Fig. SI9 in SI).

**Fig. 7:**
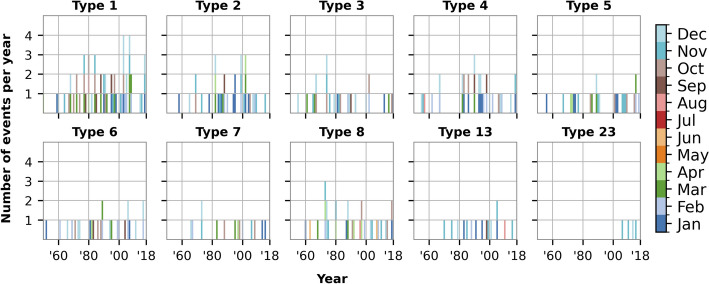
Number of events per year per event type with denoted months

The frequency analysis revealed diverse temporal behaviours among the ten event types (Fig. 7). Type 1 stood out with its relatively frequent occurrences, particularly in the 2000s, with up to four events in 2003 and 2007. Type 2 showed a more sporadic pattern but clustered in the 1980s and early 2000s, a behaviour also observed in Type 3, though on a smaller scale and with more irregularity. Type 4, while active from 1980 to the early 2000s, never exceeded three occurrences per year. Type 5 showed clustering in the 1970s, late 1980s / early 1990s, and 2000s, but remained less frequent overall. Types 6 and 8 were relatively consistent across decades but rarely exceeded one event per year. Type 7 displayed irregularity, with some grouping in the late 1990s, whereas Type 13, which, despite fewer total occurrences, maintained a steady pattern, particularly during the 1980s and 1990s. Lastly, Type 23 as the least frequent type, emerging only in the 2010s.

A notable trend was the clustering of activity for most event types from 1980 to the early 2000s. This period coincided with a predominantly positive phase of the NAO index; a climatic phenomenon associated with increased storm activity over the North Sea. To assess its potential influence, the correlation between storm surge frequency and the NAO index was calculated. A moderate positive correlation was found: Pearson’s r = 0.31 (*p* = 0.010), Spearman’s ρ = 0.33 (*p* = 0.006), and Kendall’s τ = 0.22 (*p* = 0.010). These results suggest that while the NAO may have influenced storm surge frequency, other factors were likely also contributing. Most events were observed in late fall and winter, with occasional occurrences in spring and rare instances in summer.

### Characteristics of remarkable storms

The cumulative storm surge intensity was calculated for each event in the dataset, but here we present only ten selected events.

Table 2 shows the date of peak for each storm, maximum cumulative intensity, the station where this peak intensity was recorded, event duration, total time during which the sea level was above the 99th percentile, maximum magnitude, and event type. Here, it is important to note that this study focused only on the non-tidal residual, ignoring the tidal component. Thus, the total extreme sea levels that ultimately determined the extent of coastal flooding were not considered. Additionally, this list should not be interpreted as a comprehensive record of significant events.

**Table 2:** This table highlights ten selected storm surge events. The events are ranked in descending order based on maximum cumulative intensity

	Maximum intensity (mh)	Station	Date of peak	Duration (h)	Time above 99th perc. (h)	Maximum magnitude (m)	Event type
1	120.5	Harlingen	26 Feb 1990	144.7	59.8	3.3	30
2	88.7	Harlingen	23 Dec 1954	219.0	48.0	2.9	9
3	87.3	Eemshaven	25 Jan 1993	167.3	58.0	2.1	25
4	82.8	Nes	15 Nov 1977	160.0	59.0	2.2	16
5	82.0	Eemshaven	24 Nov 1981	230.0	46.0	2.7	38
6	72.1	Harlingen	5 Jan 2012	172.5	45.7	2.4	45
7	71.1	Hoek van Holland	1 Feb 1953	222.0	36.0	3.1	49
8	65.4	Eemshaven	6 Dec 2013	120.0	29.8	3.3	23*
9	64.5	Harlingen	3 Jan 1976	221.0	30.0	3.5	18
10	48.5	Eemshaven	9 Nov 2007	262.0	28.3	2.7	5*

*Prevalent event types

**Fig. 8:**
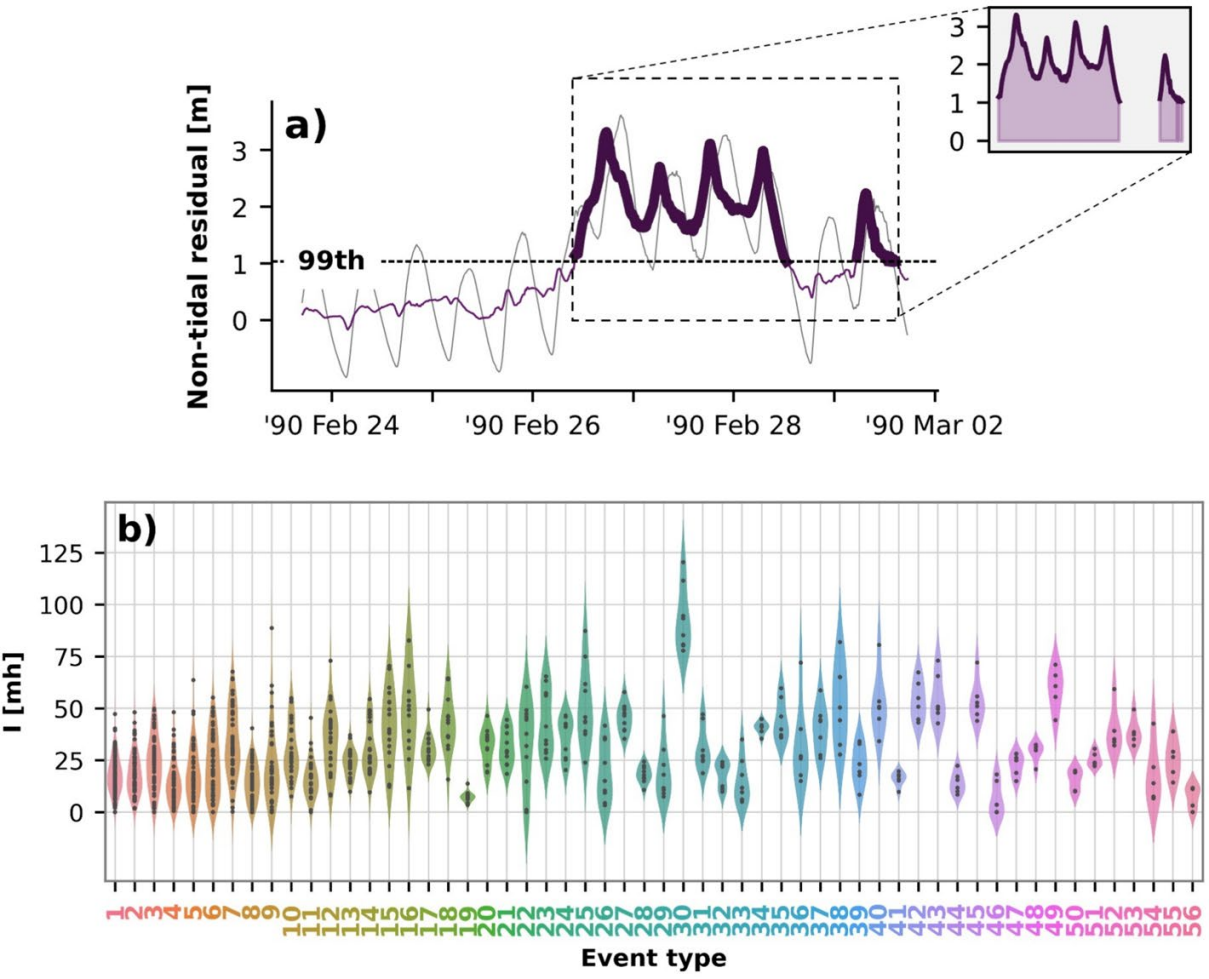
**a** Time series of the 1990 event’s storm surge (*purple*) and total observed sea level (*grey*) at Harlingen. Storm surge segments above the 99th percentile are highlighted, with a zoomed-in view where the shaded area under the curve represents the cumulative surge intensity. **b** Distributions of cumulative surge intensities per event type

The cumulative surge intensity was defined to account for both the time of the event exceeding the 99th percentile and the surge magnitude during that time. The event caused by cyclones Vivian and Wiebke in 1990 had the maximum intensity and it is illustrated in panel a of Fig. 8. The total sea level, shown in grey, exceeded 3 m, but the peak surges occurred during low/rising tides. Although historically significant, the North Sea Flood of 1953 ranked seventh on our list in terms of intensity. While all events were identified at multiple stations, the highest intensities were primarily recorded at Harlingen and Eemshaven. An exception to this was the North Sea Flood of 1953, where the peak intensity was observed at Hoek van Holland. All ten storm surge time series and the corresponding total sea levels are illustrated in Fig. SI10.

Despite the North Sea Flood of 1953’s devastating impact on the Netherlands, its surge exhibited relatively low cumulative intensity, as it was a single-peak, two-day event. More specifically, at Hoek van Holland, the maximum surge reached 3.07 m and remained above the 99th percentile for only 36 h, compared to 59.83 h during the 1990 event. Notably, the highest surge in 1953 was recorded at Harlingen, reaching 3.7 m. However, this surge occurred over a shorter duration and coincided with a low tide, unlike farther south at Hoek van Holland, where the surge contributed to an exceptionally extreme total sea level event.

Six of the ten selected historical events exhibited two distinct surges. For instance, the 1990 event, classified as Type 30, displayed two prominent surges, with multiple peaks occurring during the first surge. Similarly, the 1954 storm, categorised as Type 9, exhibited two closely spaced surges, while the 1993 storm was characterised by a short but intense initial surge, followed by a more prolonged surge two days later.

The 2013 storm surge, generated by Cyclone Xaver, was classified as Type 23, while the 2007 event caused by Cyclone Tilo fell under Type 5. Both Type 23 and Type 5 storm surges were among the prevalent types along the coast. Out of 53 time series classified under Type 5, 43 reached the 99th percentile for their respective stations, indicating that not all these surges were severe. However, the intense Nov 1977 event at Den Helder fell under Type 5. The highest magnitudes in general were recorded at northern stations. In contrast, all 10 time series under Type 23 were extreme, corresponding to the events of November 2006, November 2010, December 2013, and November 2015.

The selected events are further described in terms of their classification and their shapes can be found in Fig. 5, outlined with a dotted orange square. Type 30 events (1990 storm surge) were characterised by long durations with two distinct surges. Type 9 events (1954 storm surge) exhibited one wider or two closely spaced peaks. Type 25 (1993 storm surge) featured an initial short surge followed by a longer second surge. Type 16 also consisted of two surges, with the second being larger than the first. Type 38 displayed a small surge followed by a larger one, while Type 45 was marked by two peaks in close succession. Type 49 (North Sea Flood of 1953) has one large, distinct peak. Type 23 features a single surge, although it tends to be somewhat longer in duration. Type 18 events are characterised by a major peak followed by a smaller surge that did not reach the 99th percentile threshold, and Type 5 events had one dominant surge, sometimes with a noticeably smaller peak occurring about two days prior.

Out of these 10 types, Type 5 had the highest number of occurrences, as previously discussed in detail. Type 9 also showed a notable number of events, with some clustering in the 1980s and two separate occurrences in 2000. Type 16 was recorded five times: twice in the late 1970s, twice in the 1980s, and once in 2001. Type 23 followed closely with four separate occasions, as analysed in the previous chapter. Type 18 occurred three times, one in the mid-1970s, another in the late 1980s, and a third in the 1990s. Types 25, 38, and 49 each occurred only twice, while Types 30 and 45 were the least frequent, each appearing on just one occasion.

The distribution of cumulative surge intensity per event type is presented in Fig. 8b. Out of all event types, type 30 stood out as the one with the highest intensities. Another type with high intensity occurrences (> 75 mh), apart from the aforementioned types 9, 16, 25, and 38, was event type 40. All these types exhibited complex shapes with more than one surge. In contrast, the least intense surges (< 25 mh) were in types 19, 28, 32, 41, 44, 46, 50, and 56. Some of these types did exhibit more than one surge, however, their peaks were low. Out of the 10 prevalent types across the stations, types 7 and 23 showed the highest intensities.

## Discussion

### POT70 versus POT99

The 70th percentile threshold, while occasionally capturing more moderate sea levels, provided a significant advantage of capturing the full event evolution, rather than focusing solely on peak magnitudes. The broader selection of the hydrograph enabled the identification of secondary peaks and successive storm surges within a single event. Extreme surges can coincide with multiple factors, such as high tides, wave setup, heavy precipitation, or river runoff, all of which influence flood dynamics. A broader hydrograph ensures that their interactions and impacts can be analysed with greater accuracy, as it prevents the premature exclusion of lower peaks that may still contribute to flooding.

Therefore, the POT70 was used to extend the duration of the hydrograph, after which researchers can refine event selection using additional thresholds based on specific study objectives. In this study, event types were filtered using a threshold near the 99th percentile to focus on extreme events. However, lower thresholds may be more relevant for studying surge impacts on coastal morphology, groundwater dynamics, and beach-dune systems, while even higher thresholds may be prioritized in flood risk assessments and infrastructure resilience planning.

Moreover, a sensitivity analysis was conducted to test different thresholds (POT95, 90, 85, 80), but they failed to capture key hydrograph behaviours. For example, using the 80th percentile overlooked the negative surge in Type 8 that followed the high positive surge. A rapid drop in sea levels during such negative surges can create pressure imbalances, adding stress to waterfront structures and potentially leading to structural instability. Additionally, this post-peak behaviour is important as it distinguishes Type 8 from other event types, such as Type 1. Ultimately, the 70th percentile threshold was chosen based on the desired level of detail we wanted to preserve.

To illustrate the advantages of using a lower threshold to expand the hydrograph, two examples are provided: the Gale of January 1976, also known as the Capella storm, and the North Sea Flood of 1953.

**Fig. 9:**
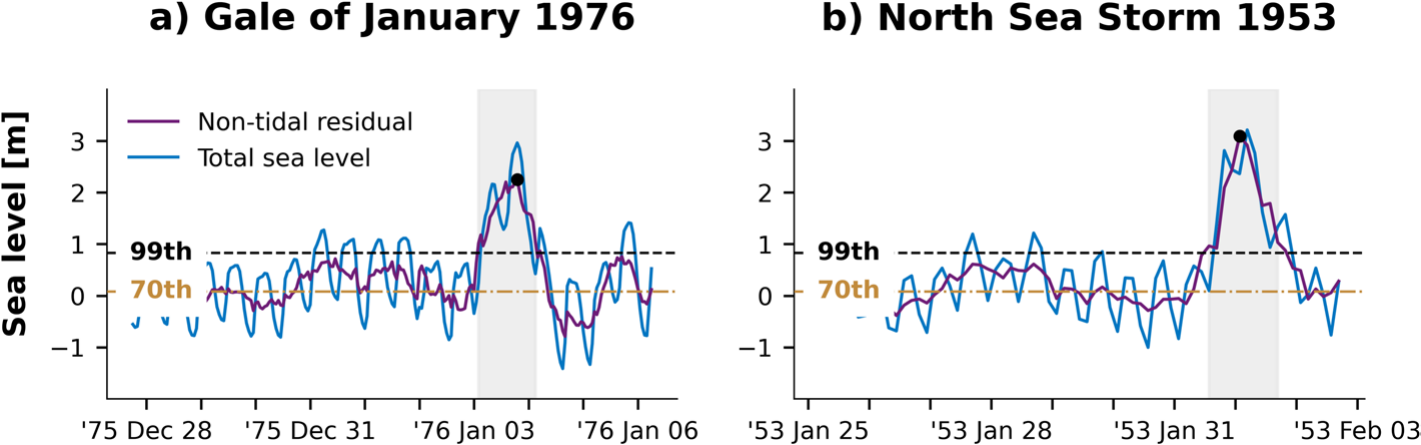
Time series analysis of storm surge events at Den Helder using different thresholds**.** Panel **a** shows results for the Gale of January 1976 and panel **b** for the North Sea Storm of 1953. The time series shows the entire duration of the event as detected using the 70th percentile threshold (POT70), while the shaded area represents the event duration identified with the 99th percentile threshold (POT99). Both the 70th and 99th percentile values as well as peak magnitudes are indicated on the graphs

For the Gale of January 1976, using the 99th percentile as the initial threshold (POT99) captured only the peak of the event, from 3 Jan 1976 at 02:00 h to 4 Jan 1976 at 03:00 h. In contrast, POT70 captured a broader time series, spanning from 27 Dec 1975 at 18:00 h to 6 Jan 1976 at 00:00 h. This broader time frame allowed for detailed observation of the entire storm surge hydrograph, as shown in panel a of Fig. 9, which shows data at Den Helder. While POT99 did record the main peak of the storm surge, it missed the elevated sea levels preceding the storm surge and did not record the smaller surge that followed the peak. Such a smaller surge, in the presence of hydraulic infrastructure, could be significant for maintenance purposes or critical if any failure happened during the main peak. It is possible that the wave before the peak was a forerunner wave, indicating the storm’s approach, while the post-peak surge could have been caused by coastally trapped waves (Munk 1947; Pattiaratchi et al. 2021; Trinh et al. 2020; Suh et al. 2018). Confirming this would require further analysis.

Similarly, panel b of Fig. 9 shows the infamous North Sea Flood of 1953. POT99 in this case captured the event more accurately, however, it again did not account for the elevated sea levels preceding the storm, possibly caused by a forerunner indicating the storm’s approach. With rising sea levels, understanding such pre-storm and post-storm surge conditions could be critical for assessing the full impact of storm surges and improving preparedness and response strategies.

### Time series clustering

Among the 56 identified types, some event types displayed a similar triangular shape, with peaks shifted in time. While it might have been possible to reduce the number of clusters by combining these types, they were kept separate in this analysis. This decision was based on variations in event durations and the possibility that differences in the physical environments across the monitoring stations contributed to distinct variations in sea level response. Although the peaks of these events may have appeared similar, the conditions before and after the peak storm surge, relative to the 70th percentile threshold, clearly differentiated them. These differences underscored the importance of considering both the timing and environmental factors that shaped each event, rather than simply focusing on peak similarity.

In some cases (e.g., Type 5), events with similar shapes but different time scales were grouped under the assumption that their surge patterns reflected comparable dynamics. However, this assumption may oversimplify the complex relationship between atmospheric conditions and sea level response. The clustering process, which treated event duration and shape equally, did not fully account for variations in meteorological forces and introduced some uncertainty. While weighting duration more heavily might have benefited the results, the decision to apply equal weighting was made to explore the potential of unsupervised learning techniques.

Therefore, it is important to recognise that this classification process inherently involves a degree of own judgment. Although the clusters could have been further merged or separated, the approach adopted in this study sought to strike a balance between data-driven insights and an understanding of complex environmental phenomena. Ultimately, this method offers valuable insights into storm surge dynamics, but it is crucial to recognize the limitations of grouping events primarily based on hydrograph shape. Future research could address these challenges by analysing how different driving mechanisms influence event clustering and surge behaviour.

### Multiple-peak events

Out of the 56 identified event types, 56% displayed more than one distinctive peak, including 40% of the 10 most prevalent types. Here, POT99 failed to capture more than one peak in 38% of the time series for Type 3, 65% for Type 4, 29% for Type 6, and 19% for Type 7. The incomplete capture highlighted the challenges in accurately documenting all peaks within the storm surge hydrograph using the 99th percentile method. Additionally, event Type 3 ranked third among the 56 types and exhibited two distinctive peaks. This observation was noteworthy because standard Dutch coast hydrographs typically did not account for events characterised by twin peaks or dual storm surge occurrences (Geerse et. al 2019). These findings indicated that two-peak events were not rare anomalies but rather a common pattern observed across the monitored sites.

Two-peak shapes were amongst the top two most common types at nearly all stations, apart from Cadzand, Lauwersoog, and Eemshaven. The absence of two-peak events at Lauwersoog and Eemshaven could be explained by their geographic and morphological characteristics along the far northeast coast, where they are shielded from the North Sea by the Frisian Islands. Additionally, the tide gauge at Lauwersoog is located near a dike that encloses the man-made lake Lauwers Sea (Dutch *Lauwersmeer*), while the Eemshaven gauge is situated in a harbour at the estuary of the river Ems. In contrast, Cadzand is located in the far south near the Westerschelde estuary and the former Zwin inlet. These geographical and structural differences likely influence wave and storm surge patterns. In Cadzand's case, the frequent north-northeast storms may lead to a weaker sea level response in the south, which could contribute to the limited occurrence of two-peak events.

The analysis further revealed that, for the four prevalent two-peak types, the second peak typically arrived about two days after the initial one. This aligns with previous findings by Dillingh et al. (1993), who reported an average interval of 2.4 days between storm surge events. However, more recent studies suggest a longer event duration. Martin et al. (2024) reported that the standard storm surge event duration along the Dutch coast is approximately four days, and Diakomopoulos et al. (2024) recommended using a four-day interval for declustering in POT analysis. The latter was based on Spearman's correlation coefficients between extreme non-tidal residuals and wind speed at Hoek van Holland. These findings suggest that the consecutive peaks observed in multi-peak events are likely not independent, but rather statistically related, and should be treated as part of the same event from a sea level perspective. Thus, fixed declustering of statistically extreme storm surge events might fail to capture the physical dependencies of consecutive surges.

The observed multiple peaks could be attributed to factors such as the tide-surge interaction, changes in atmospheric conditions (e.g., shifts in wind direction), forerunner waves indicating the storm’s arrival, coastally trapped waves, external surges that can occur simultaneously with storm surge events (Böhme et al. 2023; Fenoglio-Marc et al. 2015), river runoff, or storms clustering in time. For example, six of the ten selected historical events exhibited two distinct surges for which the historical atmospheric records revealed were likely caused by multiple low-pressure systems, which either merged or crossed paths as they moved over the North Sea. This aligns with studies showing that extra-tropical cyclones tend to cluster in time and reinforces the likelihood that some peaks are statistically dependent due to related weather systems and other physical processes (Priestley et al. 2017; Hauser et al. 2023).

### Other storm surge characteristics

In addition to identifying numerous multiple-peak events, it was observed that storm surges exceeding the 99th percentile for approximately two days exhibited characteristics similar to those with extreme sea level intervals lasting only a few hours. This variability in storm surge behaviour may be attributed to several factors, including the natural variability of the atmospheric and oceanic driving mechanisms, the timing of tidal cycles, and site-specific geomorphological features. These elements likely interact in complex ways, influencing the development and intensity of storm surges. A more in-depth investigation into these factors could enhance the precision of storm surge event characterization, allowing for better understanding and forecasting of storm surge impacts.

**Fig. 10:**
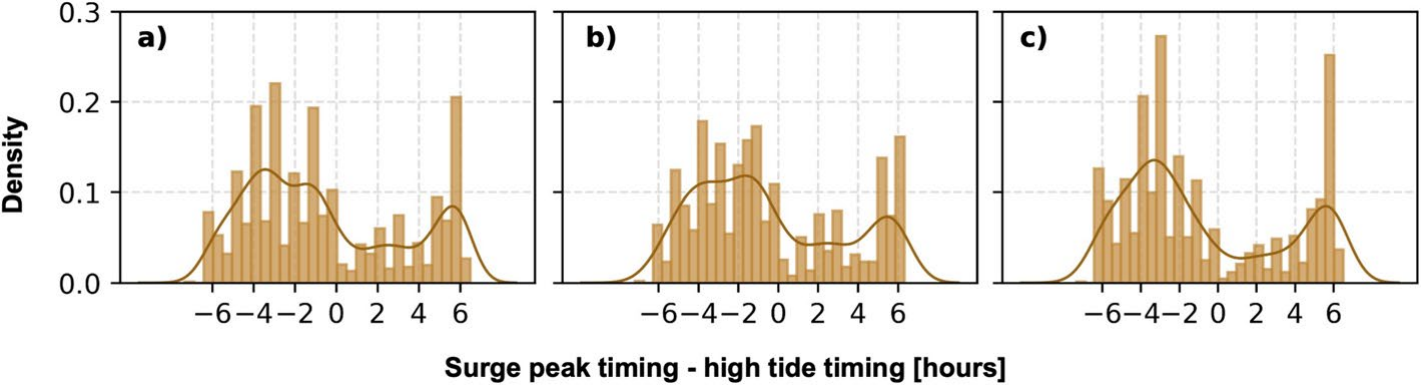
Probability density function (PDF) of the timing of the storm surge peaks with respect to the closest high tide for **a** all extracted peaks, **b** peaks between 0.5 and 1 m, and **c** peaks exceeding 1 m. The smooth line shows the PDF obtained with the Kernel Density Estimation (KDE) method, and the bars the empirical PDF

It was also noted that storm surge peaks, particularly those categorised under prevalent event types, tended to occur during low or rising tides. This is a result of tide-surge interactions in the North Sea since a positive surge increases the phase speed of both tide and surge as they travel along the coast (Horsburgh and Wilson 2007). A similar pattern was evident across all analysed time series, as shown in Fig. 10. To examine this, peaks were extracted from all 972 time series, allowing multiple peaks per event. A positive phase difference indicates that the storm surge peak occurred after the nearest high tide, whereas a negative phase difference signifies that the surge peak preceded the high tide. The analysis of all identified peaks revealed a clear tendency for surge maxima to occur during rising tides (negative phase difference) and low tides (positive phase difference), aligning with previous findings (Horsburgh and Wilson 2007; Chbab et al. 2015; Geerse et al. 2019). In shallow waters, this complex pattern was likely influenced by the distortion of higher tidal harmonics, further modifying the timing of surge peaks relative to the tide. Additionally, since wind stress is more effective at raising the sea surface in shallow water, the surge generated by wind stress is expected to be larger at low tide than at high tide (Horsburgh and Wilson 2007; Pugh 1987).

The bimodal pattern was even more pronounced for peaks exceeding 1 m, while medium peaks (0.5–1 m) showed a more dispersed distribution. Although these medium peaks still predominantly occurred during low and rising tides, more instances were occurring closer to high tide timing (-2 to 0 phase difference). This is important as even moderate surge peaks at high tide can significantly elevate total sea levels, increasing the risk of flooding. Therefore, they should not be overlooked from analyses, as they contribute to coastal vulnerability and extreme water levels. Additionally, the probability density functions (PDFs) in Fig. 10 presented a combined analysis of peaks from all stations. However, it's important to note that stations exhibit varying tidal behaviours, and even small variations in the astronomical tide could lead to significant changes in surge patterns (Geerse 2020). Furthermore, phase differences could also be influenced by local physical conditions. Future studies would benefit from investigating the phase differences at individual stations to identify spatial variations.

Another key finding was the clustering of storm surge activity between the 1980s and early 2000s, coinciding with a predominantly positive phase of the NAO index. The correlation analysis revealed a moderate positive relationship between storm surge frequency and the NAO, supporting the hypothesis that a positive NAO phase may contribute to increased storm activity over the North Sea. Other potential mechanisms were not examined, as this was outside the scope of the study. It is also possible that this clustering was influenced by the availability of more comprehensive or higher-quality data during this period, further analysis would be needed to confirm this.

The analysis of historical events showed that the 2013 storm surge, generated by Cyclone Xaver, was classified as Type 23, while the 2007 event caused by Cyclone Tilo was categorized as Type 5. Both Type 23 and Type 5 were among the prevalent types along the coast. However, the analysis showed that not all time series under Type 5 were equally severe, despite showing similar surge patterns. Additionally, the 1953 storm surge, which caused the devastating North Sea Flood, was found to have a similar shape to an event that occurred in 1965 at Harlingen, with both being classified as Type 49. This suggests that the 1965 storm produced a surge resembling the one responsible for the 1953 flood. However, it is crucial to incorporate the tidal component into the analysis to assess the potential for coastal flooding during such events. Calculating the cumulative intensity of the total sea level, rather than focusing solely on the storm surge, could yield different results due to the joint tidal and surge dynamics and provide valuable insights for future coastal flooding studies. 

Furthermore, each selected remarkable storm surge event was detected at multiple stations along the North Sea coast. However, most of the highest recorded intensities were concentrated at either Harlingen or Eemshaven, with the notable exception of the North Sea Flood of 1953, which had its maximum intensity recorded at Hoek van Holland. The maximum surge magnitude during the 1953 event, however, was again recorded at Harlingen, albeit with a shorter duration. This pattern highlighted the significance of the northern stations in terms of experiencing more intense surge events. The wider range of event duration observed at northern stations, along with the higher values of surge peak magnitudes, indicated that the driving factors in these areas may be more favourable to intense storm surges compared to those affecting southern stations. Understanding these localised differences is crucial for improving storm surge forecasting and developing targeted mitigation strategies for different regions along the North Sea coast.

## Conclusions and future recommendations

The results of this study provide insight into the characteristics and temporal patterns of historical storm surge events along the Dutch coast. The analysis focused on detecting storm surge events using two different thresholds: the 70th percentile (POT70, 0.08–0.10 m) and the 99th percentile (POT99, 0.8–1.1 m). Using a novel classification framework developed in this study, POT70 storm surges along the Dutch coast were classified into 311 event types. To focus on extreme events, this was refined to 972 time series and 56 distinct event types.

The key findings from the study are as follows:

- *Comprehensive analysis with POT70*: POT70 allowed for a more detailed analysis, effectively identifying multi-peak events, while POT99 focused on peak surges but often missed crucial pre- and post-storm activities. This distinction underscores the importance of considering a broader range of surge characteristics in coastal management and impact assessments.
- *Flexibility of POT70*: The initial classification of POT70 provides a versatile library for surge analysis, allowing researchers to adjust thresholds and refine event selection to other study objectives.
- *Two-peak events*: Two-peak events were notably common, with the second peak occurring about two days after the first, often coinciding with low or rising tides.
- *Regional variability*: The northern stations experienced stronger surges than those in the south, as indicated by the wider range of event durations and higher values of surge peak magnitudes observed in the north.
- *Surge complexity and variability*: The findings highlighted the complex and variable nature of storm surge patterns along the Dutch coast, even though some degree of judgment was applied in the classification process.
- *Challenging traditional hydrographs*: The new approach challenges the traditional triangular hydrographs, which may oversimplify surge dynamics. This is especially important as climate change introduces new uncertainties.
- *Possible applications*: The findings offer support for better-informed coastal defence designs, ensuring that flood protection measures account for a wider range of possible surge scenarios. This research lays a foundation for exploring key surge drivers, assessing existing estimation methods, and improving model validation and development. Additionally, understanding how different surge types evolve over time can support advancements in forecasting and early warning systems.
- *Cumulative storm surge intensity*: The introduction of a cumulative storm surge intensity metric offers the potential for a more holistic assessment of flood risk, which could be extended to total sea levels.
- *Adaptability*: The methodology developed in this study is adaptable to other coastal regions and could be applied even globally.

Future research should analyse the physical processes driving different event types, particularly the multiple-peak events, as not all such events necessarily result from consecutive extra-tropical cyclones. Further investigation of atmospheric, wave, and river runoff data, as well as tide-surge interactions, is recommended. Additionally, enhancing clustering analysis by incorporating spatial dimensions would provide a more integrated understanding of storm surge patterns along the entire coastline. Spatio-temporal clustering could offer deeper insights into how multiple-peak events interact along different coastal regions, providing a clearer picture of storm surge dynamics as they evolve both spatially and temporally.

## Supplementary Information

Below is the link to the electronic supplementary material.

Supplementary file1 (DOCX 18088 KB)

## Data Availability

The data will be publicly available through the 4TU.ResearchData repository (10.4121/7b9201f0-88ee-438b-8059-fddf52f66302). All global datasets used in this study are also available online, for access please refer to the corresponding references mentioned in this article. Additional data on the results can be requested from the corresponding author.

## Code availability

The code can be requested from the corresponding author.
