# Supplementary material for: Storm surge hydrographs from historical observations of sea level along the Dutch North Sea coast
Source: Nat Hazards (Dordr). 2025 May 29;121(12):14147–75. doi: 10.1007/s11069-025-07351-8 (PMC12304054; doi:10.1007/s11069-025-07351-8)
Supplement: Supplementary file 1 — Supplementary file1 (DOCX 18088 KB) [file 11069_2025_7351_MOESM1_ESM.docx]

**Storm surge hydrographs from historical observations of sea level along the Dutch Coast in the North Sea**

Mia Pupić Vurilj^1*^, José A. A. Antolínez^1^, Sanne Muis^2,3^, Oswaldo Morales Napoles^1^

^1^Delft University of Technology, Department of Hydraulic Engineering, Delft, Netherlands

^2^Vrije Universiteit Amsterdam, Institute for Environmental Studies, Amsterdam, Netherlands

^3^Deltares, Hydrodynamics and Forecasting Department, Delft, Netherlands

*Corresponding author. E-mail: [m.pupicvurilj@tudelft.nl](mailto:m.pupicvurilj@tudelft.nl), ORCHID: <https://orcid.org/0009-0003-8719-3182>;

Contributing authors: [j.a.a.antolinez@tudelft.nl](mailto:j.a.a.antolinez@tudelft.nl) (<https://orcid.org/0000-0002-0694-4817>); [sanne.muis@vu.nl](mailto:sanne.muis@vu.nl) (<https://orcid.org/0000-0002-8145-0171>); [o.moralesnapoles@tudelft.nl](mailto:o.moralesnapoles@tudelft.nl) (<https://orcid.org/0000-0002-6764-4674>)

# Supplementary Information (SI)

**UTide**

Python package used on the detrended total sea level with irregular time steps. *Method* chosen was ordinary least squares (*ols*), *nodal* parameter was set to *True* to include the nodal/satellite corrections, *trend* was set to *False* since we removed the linear trend, and a *Monte-Carlo* simulation was used for the confidence interval (conf_int = 'MC').

**Resampling in time**

Distance-based clustering, such as K-medians, is a vector quantisation method that requires a constant number of dimensions. Therefore, the storm surge time series need to have the same number of time steps as they are used as features in clustering. Since the events in the dataset have varying time steps (∆t) and durations (D), it was necessary to normalise and interpolate the time series in time to allow for comparison of the different event shapes and durations. A different approach could have been to add filling-values to match the maximum length size, experience showed that adding a “filling-value” in reef profile shape classification, biases results (Scott et al. 2020).

To resample the time series, all events were interpolated to a regular spacing ranging from 0 to 1, using the maximum value of ∆t ∙ D found across all events as the new resolution. The new time step was calculated as:

$$\begin{aligned} \Delta t_{new}=\frac{1}{\max\left( \Delta t \cdot D \right)}\#(1) \end{aligned}$$

This ensured that the event with the largest time step and duration combination served as the reference for interpolation, accommodating the varying temporal resolutions and durations of the events. Linear interpolation was applied to align data points with the new time steps. Therefore, in normalised time (from 0 to 1, a total of 1125 steps), the $\Delta t_{new}$ between time points was the same for all events (1/1125). In real-world time, the time step varied for each event due to differences in event duration. For example, an event lasting 100 hours had an interpolated time step of 100h/1125 = 0.089h, while an event lasting 40 hours had a step of 40h/1125 = 0.035h. As a result, long and short event shapes were directly compared during clustering. An example is provided in Fig. SI1 where panel a) shows a long event and panel b) shows a relatively short event. The two shapes are directly compared and not centred around the peak.


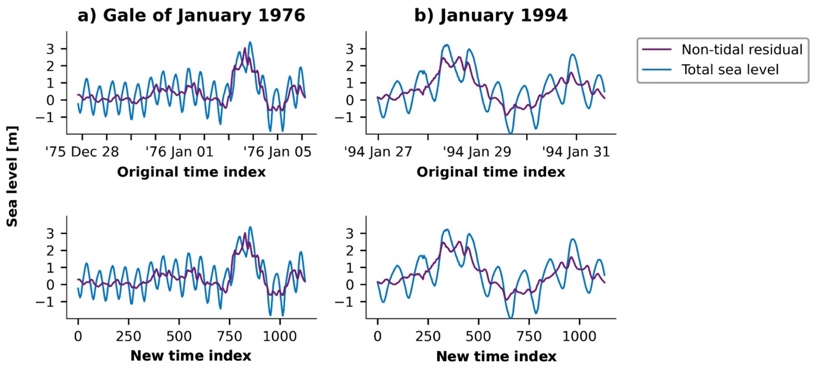


**Fig. SI1** Storm surge time series before and after resampling in time for **(a)** Gale of January 1976, representing a long-duration event, and **(b)** an event from January 1994 representing a short event

**Feature Augmentation, Standardisation, Variance Weighting & Principal Component Analysis**

We classify based on surge values (height), time series shape, and duration. Surge values (height) are the primary feature used for clustering time series shapes. A schematic overview of clustering features is provided in Fig. SI2.


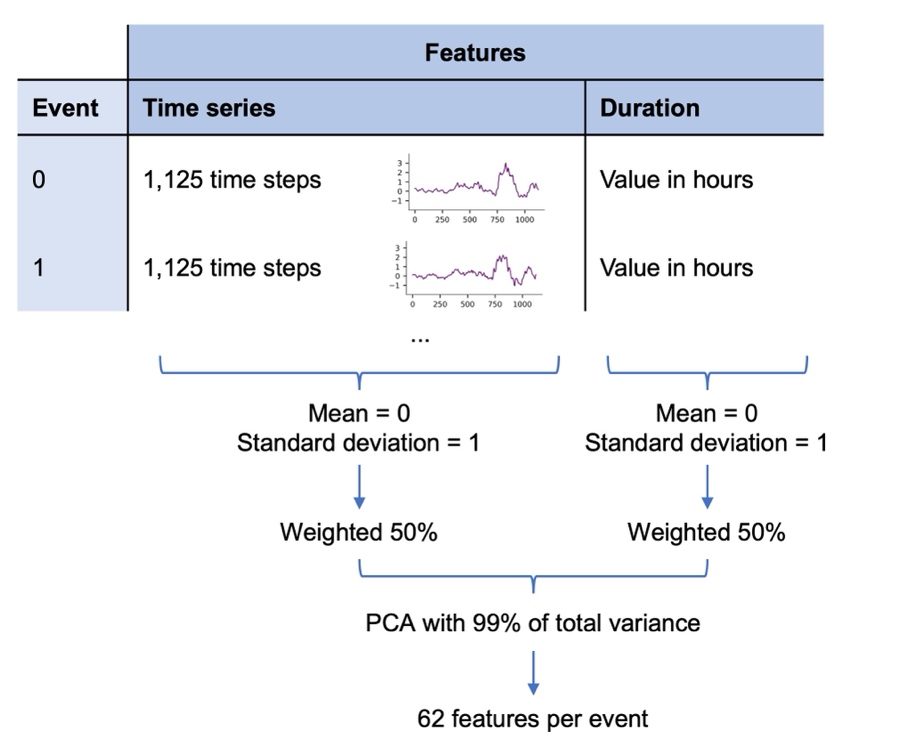


**Fig. SI2** A schematic overview of clustering features

Weighting ensures that both the time series and duration contribute equally (50%-50%) to further calculations, despite their different scales. After applying these weights, we perform Principal Component Analysis (PCA) on the combined array of weighted (1,126) features, retaining 99% of the variance.

**K-medians**

The K-medians algorithm partitions a set of points $X=\left\{ x_{1},x_{2},\ldots,x_{n} \right\} \subseteq\mathbb{R}^{d}$ into $K$ clusters $C=\left\{ C_{1},C_{2},\ldots C_{K} \right\}$ by minimising the distance of the points to the centres of the clusters (*cluster centroids*). The centres form a set of medians $M=\left\{ m_{1},m_{2},\ldots,m_{K} \right\}$, and the objective function to be minimised is defined as

$$\begin{aligned} Q\left( C,M \right)=\sum_{i=1}^{K} \sum_{x\in C_{i}} \left\| x-m_{i} \right\|\#(2) \end{aligned}$$

where $\left\| x-m_{i} \right\|$ denotes the distance between a point $x$and the median $m_{i}$ of cluster $C_{i}$. Typically, the Manhattan distance (or $L_{1}$​-norm) is used to measure this distance. (Moshkovitz et al. 2020; Whelan et al. 2015)

The algorithm starts by partitioning the input points into *K* initial sets and then calculates the centroid of each set. It then constructs a new partition by associating each point with the closest centroid. The median of each cluster is then updated by calculating the median of all the observations assigned to it. This process is iteratively repeated until convergence, which is defined as no further changes in the assignment of observations to clusters. Similar to K-means, K-medians clusters data by finding central points, but instead of using the mean to calculate cluster centres, it uses the median of each feature. K-medians is more robust and less sensitive to noise than K-means (Wittek 2014).

**OPTICS**

OPTICS (Ordering Points to Identify the Clustering Structure) is a density-based clustering algorithm that can extract clusters of varying densities and shapes as it does not require setting the number of clusters in advance (Ankerst et al. 1999). The key concept behind OPTICS is to extract the clustering structure of a dataset by identifying the density-connected points. Several core concepts define the algorithm:

**Neighbourhood radius (ε)**: This parameter defines the search radius around each data point. In OPTICS, ε is generally set to a large value, as its primary role is to ensure all possible neighbours are included in the search, rather than to restrict cluster formation.

**MinPts**: This parameter defines the minimum number of points required within the ε-radius to consider a region dense enough to be part of a cluster. MinPts helps determine the minimum density threshold for clusters.

**Core distance (ε’)**: For any point, the core distance is the minimum radius needed to capture MinPts points in the neighbourhood. If a point does not meet the minimum density (fewer than MinPts neighbours within ε), it is not considered a core point. Core distances are crucial for identifying cluster structure since they help prioritize denser regions.

**Reachability distance**: For a point *p* and a point *o*, the reachability distance from *p* to *o* is the maximum of the core distance of *p* and the actual distance from *p* to *o*. This helps in understanding the density relationship between points.

$$\begin{aligned} Reachability distance\left( o,p \right)= max\left( Core distance\left( p \right), Euclidean Distance\left( p,o \right) \right)\#(3) \end{aligned}$$

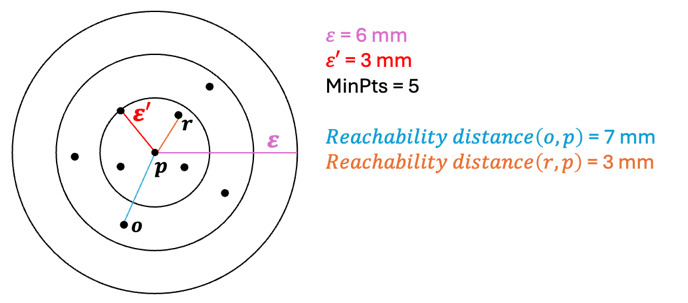


**Fig. SI3** Schematic explanation of the OPTICS algorithm

OPTICS algorithm is an iterative process with the following main steps:

1. Set an initial point and determine its core distance if it has at least MinPts neighbours within the ε radius.
2. For each point, calculate its core distance ε’.
   1. If the core distance is undefined (meaning it has fewer than MinPts neighbours within ε), that point is marked as noise, and OPTICS moves to the next point
   2. If the core distance is defined, OPTICS begins an ordering process for points, which results in the reachability plot
3. For each point *p*, update its reachability distance for each neighbouring point in the dataset.
4. Order the points based on their reachability distance and create the reachability plot.
5. Extract clusters from the reachability plot by grouping points close to each other and with similar reachability distances.

**Peak detection**


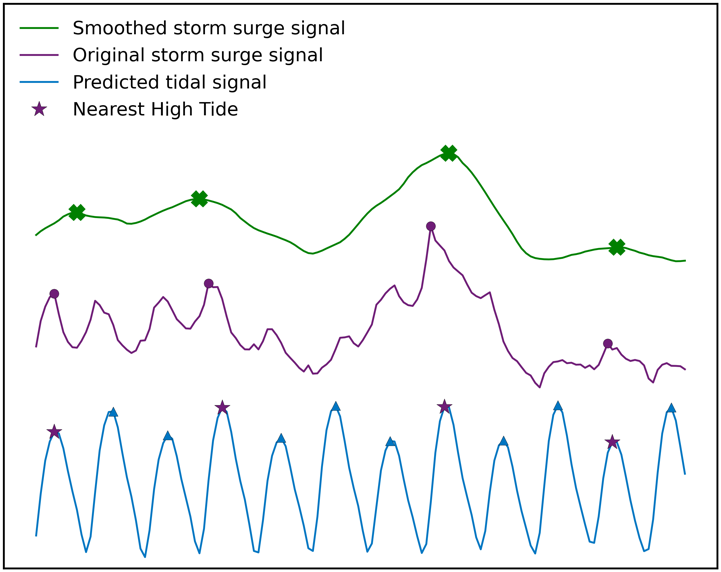
**Fig. SI4** An example of the smoothed storm surge signal (*top*), its original version (*middle*), and the simulated tidal signal (*bottom*). The corresponding peaks are shown for each signal. The high tides that are closest to the peaks identified in the original storm surge signal are highlighted with a *star*

**Distributions of durations and event’s peak magnitudes per station**


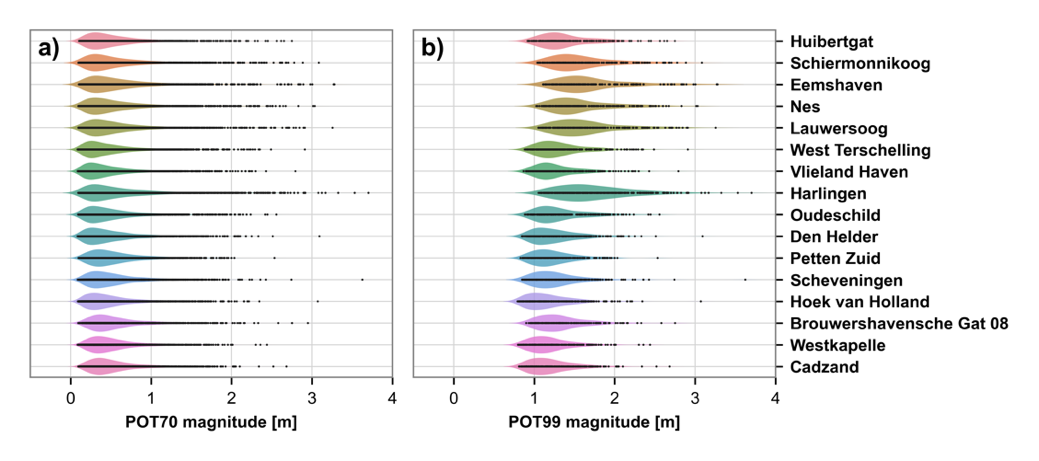


**Fig. SI5** Distributions of storm surge event’s peak magnitudes per station for **a)** POT70 and **b)** POT99


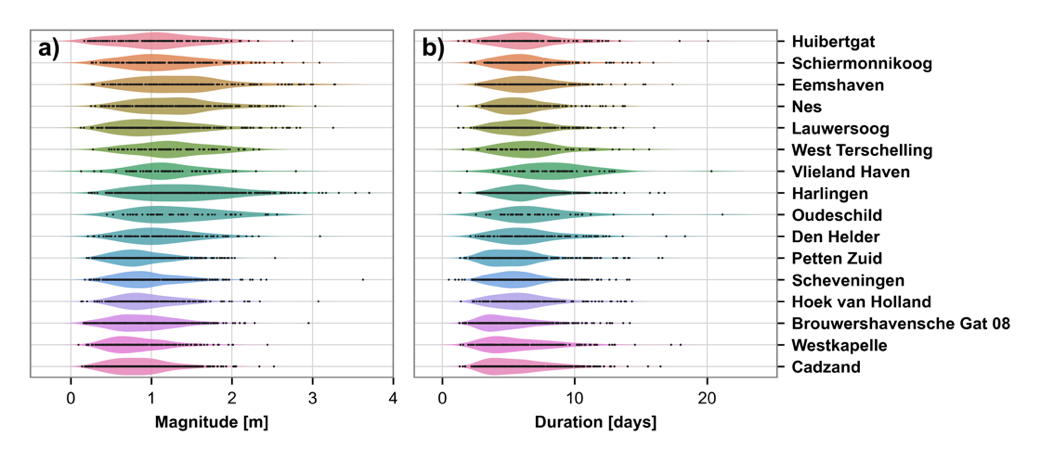


**Fig. SI6** Distributions of **a)** storm surge event’s peak magnitudes and **b)** durations per station for the MDA subset

**Characteristics of all 56 event types**


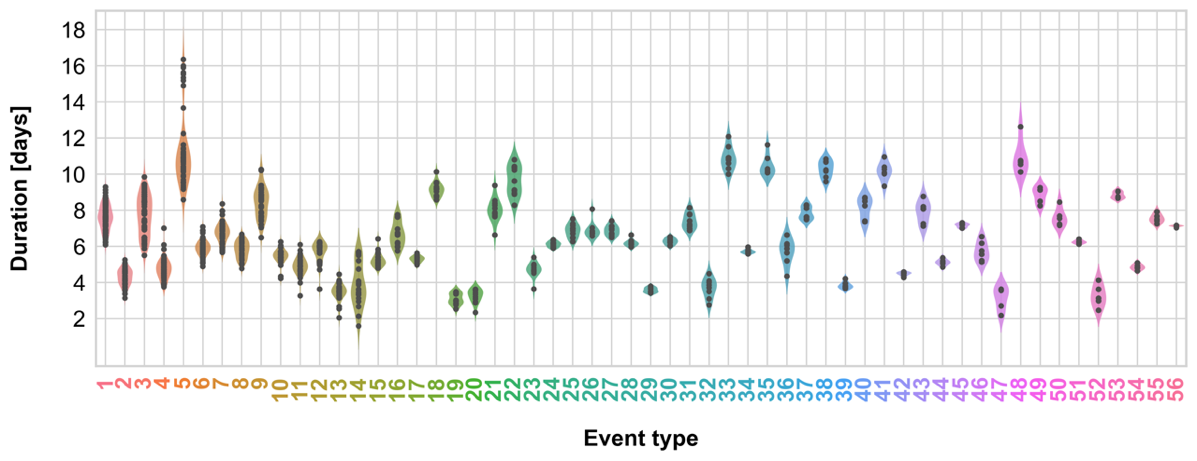


**Fig. SI7** Distributions of storm surge event durations per event type


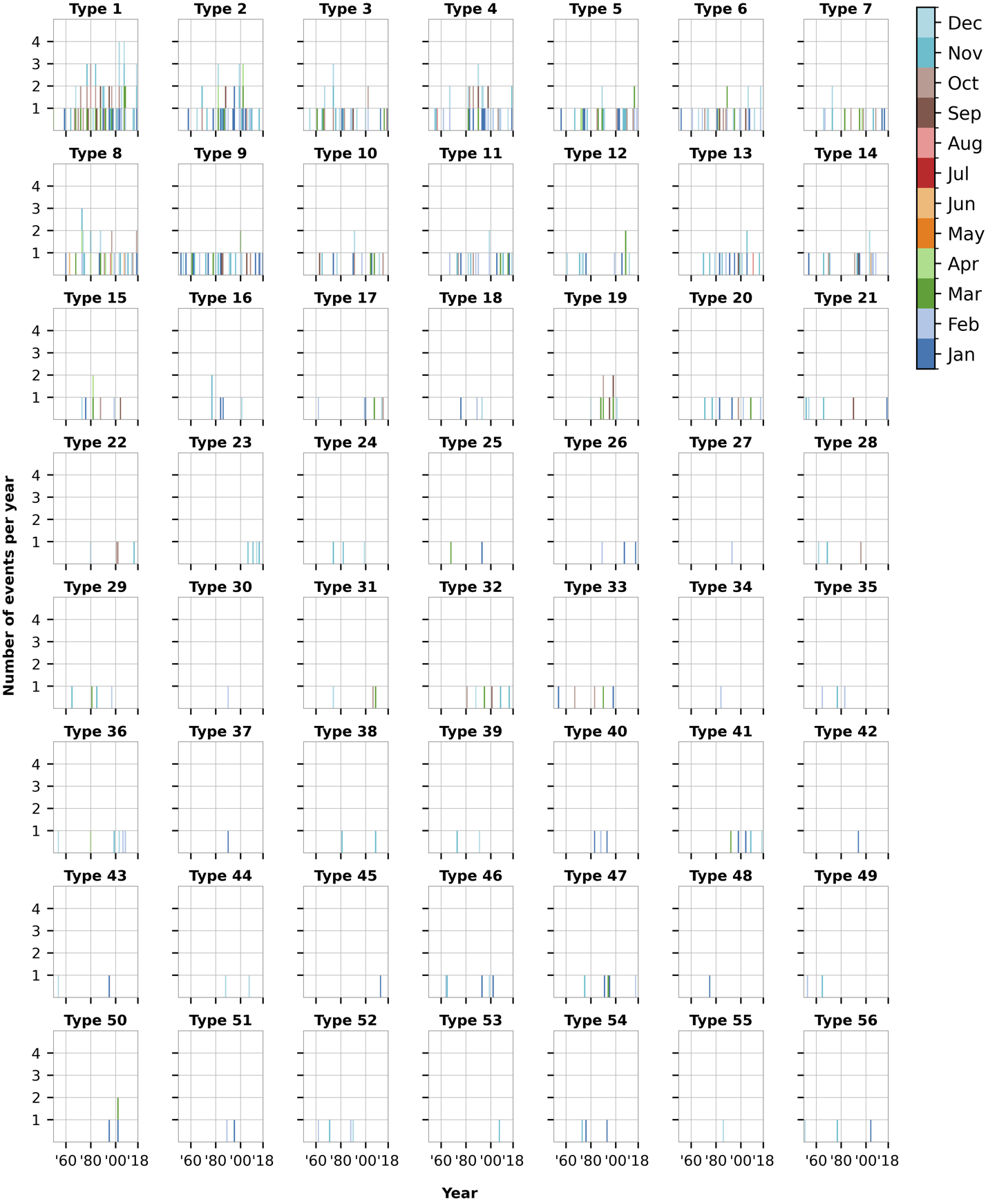


**Fig. SI8** Yearly frequency of storm surge events per type with denoted months

**Timing of peaks with respect to the closest high tide in prevalent two-peak event types**

**
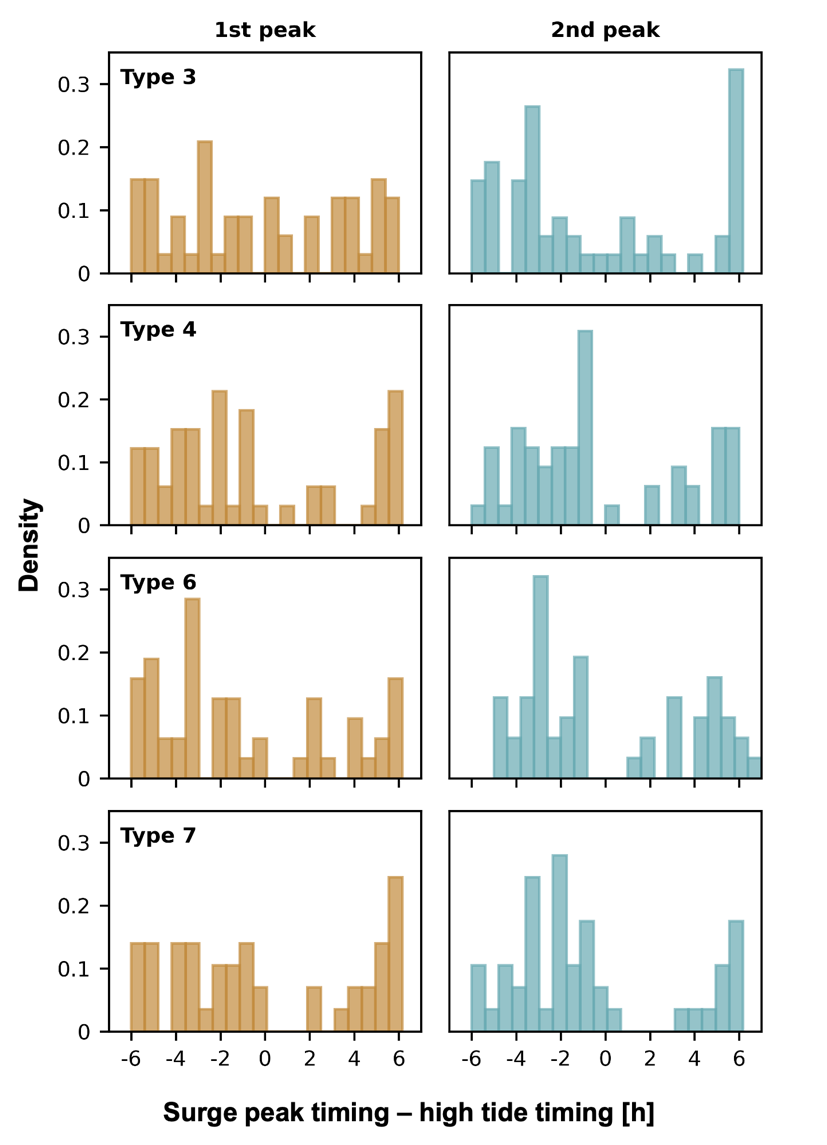
**

**Fig. SI9** Timing of peaks with respect to the closest high tide in prevalent two-peak event types

**Remarkable events**

**
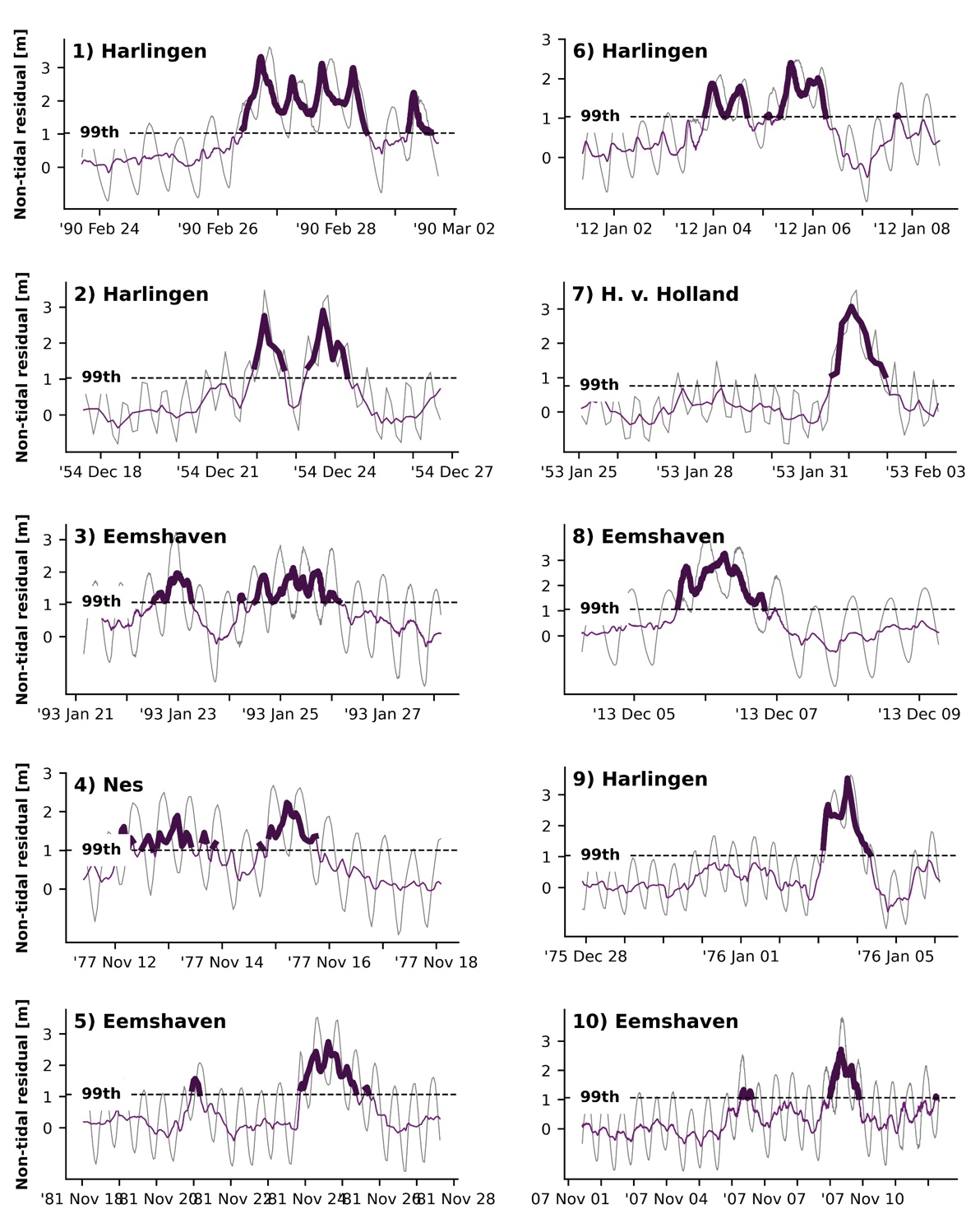
**

**Fig. SI10** Time series of storm surge (purple) and total sea level (grey) for 10 significant historical events, highlighting segments that exceed the 99th percentile surge threshold. Events are ranked by cumulative surge intensity, with the indicated station of maximum occurrence

**References**

Ankerst M, Breunig MM, Kriegel H-P, Sander J (1999) OPTICS: ordering points to identify the clustering structure. SIGMOD Rec 28:49–60. <https://doi.org/10.1145/304181.304187>

Moshkovitz M, Dasgupta S, Rashtchian C, Frost N (2020) Explainable k-Means and k-Medians Clustering. Proceedings of the 37th International Conference on Machine Learning. PMLR, pp 7055–7065

Scott F, Antolinez JAA, McCall R, et al (2020) Hydro-Morphological Characterization of Coral Reefs for Wave Runup Prediction. Front Mar Sci 7. <https://doi.org/10.3389/fmars.2020.00361>

Whelan C, Harrell G, Wang J (2015) Understanding the K-Medians Problem. Proceedings of the International

Conference on Scientific Computing (CSC). The Steering Committee of The World Congress in Computer Science,

Computer Engineering and Applied Computing (WorldComp), pp. 219-222

Wittek P (2014) 5 - Unsupervised Learning. In: Wittek P (ed) Quantum Machine Learning. Academic Press, Boston, pp 57–62
